# GluClR-mediated inhibitory postsynaptic currents reveal targets for ivermectin and potential mechanisms of ivermectin resistance

**DOI:** 10.1371/journal.ppat.1007570

**Published:** 2019-01-29

**Authors:** Mohammed Atif, Jennifer J. Smith, Argel Estrada-Mondragon, Xue Xiao, Angela A. Salim, Robert J. Capon, Joseph W. Lynch, Angelo Keramidas

**Affiliations:** 1 Queensland Brain Institute, The University of Queensland, Brisbane, Australia; 2 Institute for Molecular Bioscience, The University of Queensland, Brisbane, Australia; University of Georgia, UNITED STATES

## Abstract

Glutamate-gated chloride channel receptors (GluClRs) mediate inhibitory neurotransmission at invertebrate synapses and are primary targets of parasites that impact drastically on agriculture and human health. Ivermectin (IVM) is a broad-spectrum pesticide that binds and potentiates GluClR activity. Resistance to IVM is a major economic and health concern, but the molecular and synaptic mechanisms of resistance are ill-defined. Here we focus on GluClRs of the agricultural endoparasite, *Haemonchus contortus*. We demonstrate that IVM potentiates inhibitory input by inducing a tonic current that plateaus over 15 minutes and by enhancing post-synaptic current peak amplitude and decay times. We further demonstrate that IVM greatly enhances the active durations of single receptors. These effects are greatly attenuated when endogenous IVM-insensitive subunits are incorporated into GluClRs, suggesting a mechanism of IVM resistance that does not affect glutamate sensitivity. We discovered functional groups of IVM that contribute to tuning its potency at different isoforms and show that the dominant mode of access of IVM is via the cell membrane to the receptor.

## Author summary

Glutamate-gated chloride channel receptors (GluClRs) mediate chemoelectric inhibition in invertebrate animals and are targets for broad-spectrum pesticides such as ivermectin. However, resistance to ivermectin threatens the effective control of invertebrates that cause a range of agricultural and human diseases. This study investigates different isoforms of GluClR expressed by the major agricultural endoparasite, *Haemonchus contortus*, on a synaptic and single receptor level. We discovered that ivermectin enhances synaptic current amplitude and decay and lengthens single receptor activity. Furthermore, ivermectin is less efficacious at GluClRs that incorporate a naturally ivermectin-resistant subunit, suggesting a potential resistance mechanism. Finally, we identify two chemical interactions between the GluClR and ivermectin that determine its potency and show that ivermectin binds to GluClRs via cell membrane interactions.

## Introduction

Glutamate-gated chloride channel receptors (GluClRs) are a major class of pentameric ligand gated ion channels (pLGICs) [1] that mediate neuronal and muscular inhibition [2, 3]. They are expressed exclusively in invertebrates, making them ideal targets for the development of pesticides with minimal risk of unwanted activity at vertebrate pLGICs. GluClRs are of interest because they are an excellent model for understanding pLGIC structure at high resolution [4, 5], function and pharmacology [6, 7], and because many invertebrates that express them are commercially important pest species in agriculture [8–10], aquaculture [11] and in veterinary and human health [12].

**Fig 1:**
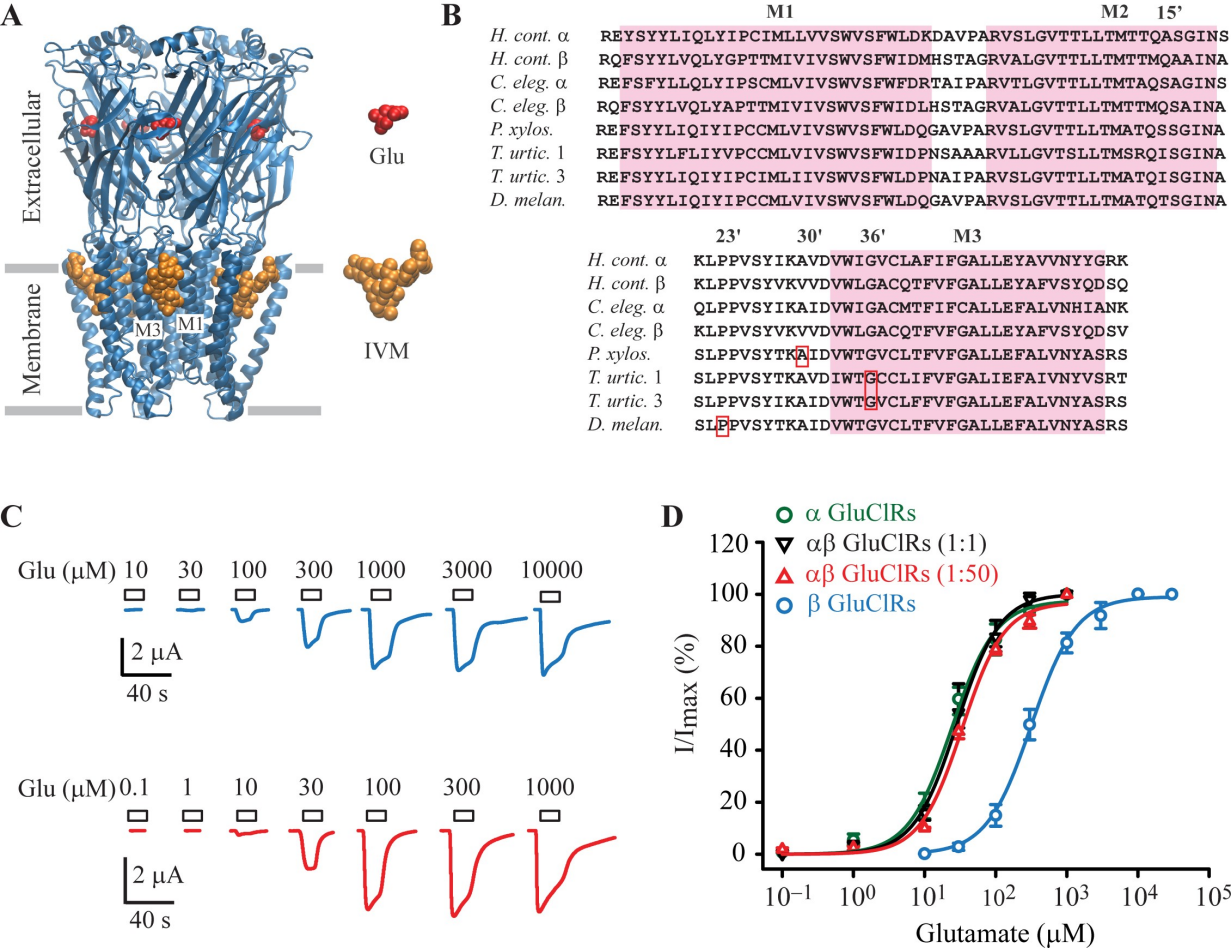
Activity of glutamate and IVM at homo- and heteromeric GluClRs. **A.** Structure of the GluClR (3RIF, [5]) showing the large extracellular domain with glutamate bound at interfaces between adjacent subunits. Also shown is the transmembrane domain, which consists of four helices (M1-M1) per subunit and IVM bound between the M1 and M3 of adjacent subunits. **B.** Sequence alignments of selected invertebrate GluClRs subunits showing the IVM binding site, which consists of the first (M1) to third (M3) transmembrane domains. Boxed in red are sites of missense mutations that reduce IVM sensitivity. Abbreviations are *H*. *cont*., *H*. *contortus*, *C*. *eleg*., *C elegans*, *P*. *xylos*., *P*. *xylostella*, *T*. *urtic*., *T*. *urticae (isoforms 1 and 3) and D*. *melan*., *D*. *melanogaster*. **C.** Example currents obtained in oocytes injected with the β subunit (above) or the α and β subunits (ratio 1:50, below) in response to the indicated glutamate concentrations. **D.** Group concentration-response data for the indicated GluClRs. Holding potential was –40 mV.

Ivermectin (IVM) is a highly effective, broad-spectrum anthelminthic drug that targets GluClRs. Its binding site is formed between the first (M1) and third (M3) transmembrane domains contributed by adjacent subunits [5] (Fig 1A and 1B). The drug is used extensively as an insecticide and anti-parasitic agent [13], where its mechanisms of action include paralysis of feeding structures, such as the pharynx, in adult organisms [14–16] and impairment of larval viability [13, 17]. However, resistance to IVM and related macrocyclic lactones, such as abamectin, is an emerging concern that threatens food production [9, 18] as well as human [19] and animal health [20] on a global scale. Underscoring the imperative for a greater understanding of the molecular targets of pesticides and mechanisms of resistance, studies predict that increasing seasonal temperatures due to climate warming will significantly exacerbate agricultural loss that results from an increase in populations of herbivorous pests [10, 21] and veterinary parasites [9]. The chemical synthesis of new IVM derivatives is a key strategy in overcoming IVM resistance as it has the potential to probe specific molecular groups of IVM [22] and their interactions with IVM sensitive and resistant forms of GluClR.

Wild, IVM-resistant pest isolates that express missense point mutations to GluClR subunits have been identified in the herbivorous species, *Plutella xylostella* [23, 24] (A30’V) and *Tetranychus urticae* [25–27] (G36’D, G36’E) and in a laboratory-induced mutation to a GluClR subunit of *Drosophila melanogaster* (P23’S) upon exposure to IVM [28] (Fig 1B). Point mutations to GluClR subunits are also being reported in human head lice, *Pediculus humanus*, that include the A55’V, and S46P and H272R, the latter two mutations being located in the extracellular and intracellular domains of the receptor, respectively [29].

In addition to mutations, the sensitivity of GluClRs to IVM is affected by subunit composition. Cully et al. 1994 first identified GluClR subunit isoforms in the nematode, *Caenorhabditis elegans* and demonstrated that they could assemble as homo- and heteromeric receptors with different pharmacological properties [1]. *C*. *elegans* expresses a β subunit that is insensitive to IVM when expressed as a homopentamer, and acts to reduce IVM sensitivity when co-assembled with the IVM-sensitive α subunit [1] (Fig 1B). A naturally IVM-resistant variant of an α subunit isoform has also been discovered in a strain of *C*. *elegans*. IVM insensitivity can be overcome by overexpressing IVM-sensitive subunits in the resistant *C*. *elegans* strain [30]. Moreover, IVM effectively kills the larvae of the human pathogens, *Onchocerca volvulus* and *Wuchereria bancrofti*, but is less effective against the adult stages of these organisms [13], suggesting a developmental switch in GluClR subunit composition. Evidence for selection pressure on GluClR isoform expression that confers IVM insensitivity comes from IVM-resistant isolates of the bovine pathogens, *Cooperia oncophora* and *Ostertagia ostertagi*, which demonstrate transcriptional downregulation of genes that encode IVM-sensitive GluClR subunits [31].

*H*. *contortus* is one of the world’s most economically important agricultural parasites that infects domestic ruminant animals, and field resistance to IVM is well-documented [32]. It has also been reported to infect wild ruminants, including species that are critically endangered [33]. This endoparasitic nematode has six GluClR genes encoding at least eight subunits [34]. Functional expression of wild-type homomeric GluClRs comprising α (avr-14b) subunits have been reported previously, including mutations to this subunit that greatly reduce IVM sensitivity [6, 35, 36]. Although the β subunit of *H*. *contortus* was shown to have overlapping distribution patterns with the α (avr-14b) and other α subunit isoforms [14], little is known about its function. Notably, it is not known if the β subunit forms functional homomeric receptors or whether it can combine with other subunits to form heteromers. It also remains to be determined which homomeric or heteromeric combinations of α and β subunits cluster at postsynaptic sites to mediate inhibitory postsynaptic currents (IPSCs). This deficiency represents an obstacle to understanding basic invertebrate neurobiology and determining the effects of drugs on neuronal and muscular inhibitory input, including highly lipophilic drugs, such as IVM that exhibit quasi-irreversible effects on GluClRs [37]. Due to its lipophilic nature, IVM is believed to diffuse through the body cuticle of nematodes [38] and partition into cell membranes of target organisms where it reaches a high local concentration [39]. We have recently shown that single receptor active periods of homomeric α (avr-14b) GluClRs (α GluClRs) increase in duration over 1–2 minutes after exposure to IVM, suggesting that the drug equilibrates in the membrane over time to produce maximum receptor potentiation [35].

In this study, our aims were to determine (1) whether the β subunit of *H*. *contortus* can assemble as both β homomeric and αβ heteromeric GluClRs (β GluClRs and αβ GluClRs), (2) whether subunit composition determines IVM sensitivity, (3) the potency of synthetically modified analogues of IVM (4) whether β and αβ GluClRs mediate IPSCs with different properties, and finally, (5) whether the membrane partitioning and diffusion properties of IVM correspond to the time-course of current potentiation.

## Results

### GluClRs comprising homo- and heteromeric combinations of α and β subunits of *H*. *contortus*

To test for functional expression of β and αβ GluClRs, cDNA encoding the β subunit was injected into oocytes either alone or with cDNA encoding the α subunit, at a ratio of (α:β) 1:1 or 1:50. Example glutamate-gated currents obtained from oocytes injected with the β subunit alone and the α and β subunits at a ratio of 1:50 are shown in Fig 1C. These data clearly demonstrate that the β subunit can indeed form functional β GluClRs and, given the differential glutamate sensitivity of co-injected oocytes, can assemble with the α subunit to form αβ GluClRs. The maximal currents for the α, αβ and β GluClRs were (in μA) 3.7 ± 0.8, 2.0 ± 0.4 (1:1), 2.2 ± 0.4 (1:50) and 1.9 ± 0.3, respectively and were not statistically different. Complete glutamate concentration-response experiments were done in oocytes injected with the either cDNA encoding the α or β subunit and in oocytes co-injected with both cDNAs at ratios of 1:1 and 1:50 (Fig 1D). These data show the β GluClRs are substantially less sensitive to glutamate with an EC_50_ of 394 μM compared to α homomers and the αβ heteromers (p < 0.001), all of which exhibited similar glutamate sensitivities, with EC_50_s of 28 μM (α homomers), 40 μM (αβ, 1:1) and 44 μM (αβ, 1:50) (Table 1). Notably, the fitted concentration-response plots for both heteromeric combinations of receptors did not exhibit inflections typical of mixtures of distinct receptor stoichiometries with differential EC_50_s, as has been shown for GABA-gated pLGICs [40]. These data suggest that co-injected oocytes express mostly homogeneous populations of heteromeric receptors, and that a given injection ratio gives rise to a particular homogeneous stoichiometry.

**Table 1:** Glutamate and IVM concentration-response parameters.

Ligand	GluClR	EC_50_ (nM)	Hill co-efficient	n
Glutamate	α	28 ± 6	1.9 ± 0.3	10
	αβ (1:1)	40 ± 6	1.7 ± 0.1	6
	αβ (1:50)	44 ± 3	1.7 ± 0.1	13
	β	394 ± 57***	1.5 ± 0.1	8
IVM	α	22 ± 3	1.9 ± 0.1	11
	αβ (1:1)	86 ± 14**^#^	1.0 ± 0.1	6
	αβ (1:50)	141 ± 11***	1.6 ± 0.1	14
	β	>10 μM	‐	6

One-way ANOVA **p < 0.01,

***p < 0.001 compared to α GluClRs,

^#^ p < 0.05 compared to αβ (1:50) GluClRs. cDNA injection ratios in parentheses.

**Fig 2:**
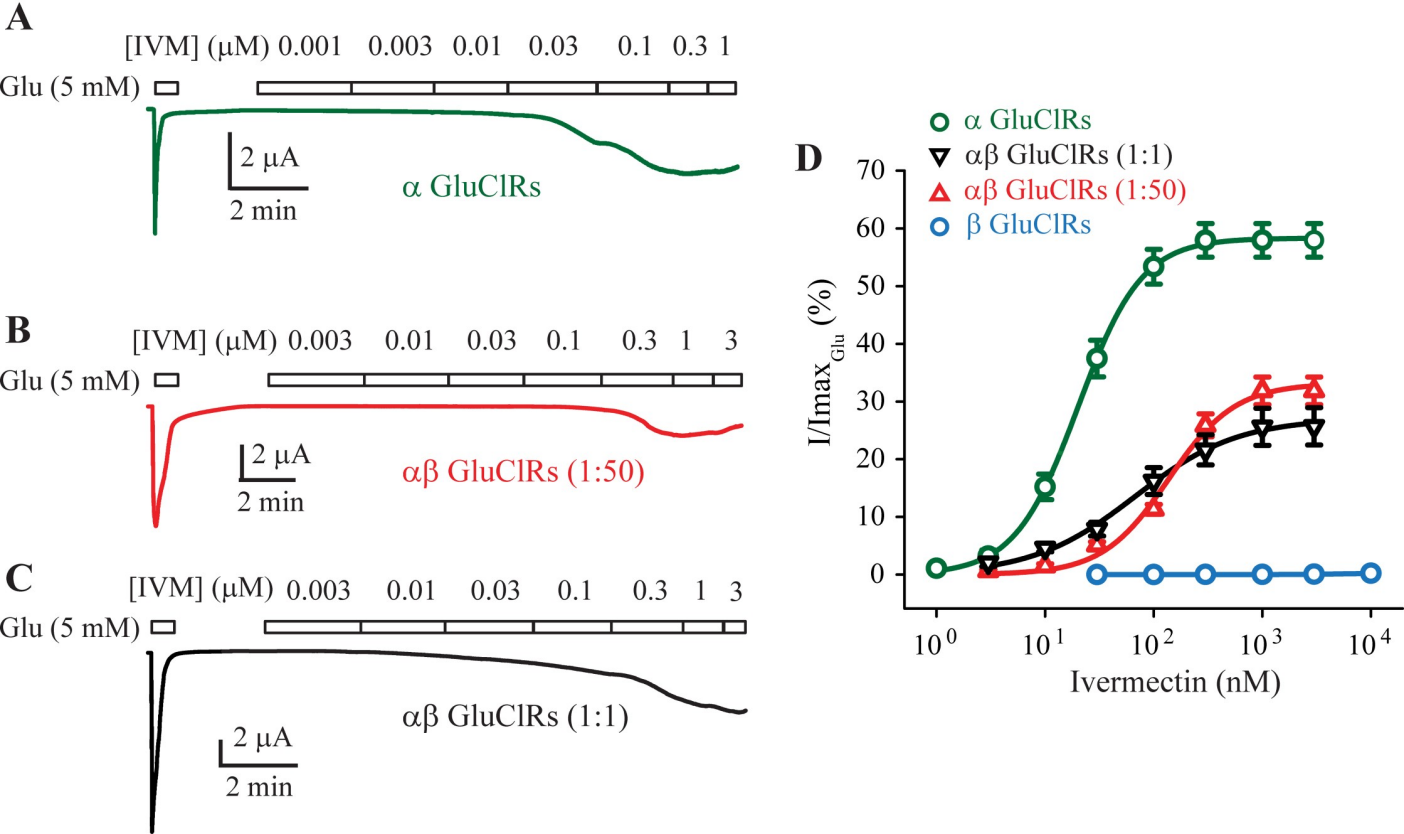
Activity of IVM at homo- and heteromeric GluClRs. **(A-C)** Representative concentration-response experiments at the indicated IVM concentrations in oocytes. IVM-induced currents were normalised to the response elicited by a saturating concentration of glutamate (5 mM) for oocytes injected with cDNA encoding the α subunit (**A)** or α and β subunits at a ratio of 1:50 (**B)** or 1:1 **(C)**. **D.** Group concentration-response data for the indicated GluClRs. Holding potential was −40 mV.

IVM concentration-response experiments were carried out to obtain additional evidence of populations of receptors that were a function of the oocyte injection ratio. Example IVM-induced currents for α GluClRs and αβ GluClRs at both injection ratios are shown in Fig 2A–2C. The corresponding group concentration-response data for four combinations of GluClRs are shown in Fig 2D. These plots indicate that α GluClRs are highly sensitive to IVM with an EC_50_ of 22 nM and a maximal response equivalent to 59% of that seen with saturating glutamate. Conversely, β GluClRs were effectively insensitive to IVM with an EC_50_ > 10 μm. The heteromeric combinations exhibited intermediate IVM sensitivities with the 1:1 injected oocytes being more sensitive (EC_50_ of 86 nM, 26% of saturating glutamate response) than the 1:50 injected oocytes (EC_50_ of 141 nM, 33% of saturating glutamate response). The % of saturating glutamate response was not significantly different between the two injection ratios. However, the EC_50_s for the two heteromeric combinations were statistically different from the α GluClRs and from each other (Table 1).

Together these data demonstrate that β subunits of *H*. *contortus* and *C*. *elegans* [1] exhibit some functional similarities. They both form homomeric receptors that are insensitive to IVM and can combine with α subunits to reduce IVM sensitivity. However, unlike the β GluClRs of *C*. *elegans*, those of *H*. *contortus* are responsive to glutamate, albeit with a reduced sensitivity. The data also show that the β subunit can co-assemble with the α subunit without reducing the sensitivity of the receptors to the neurotransmitter, glutamate. Furthermore, IVM potency is reduced when oocytes are injected with an excess of β subunit cDNA, implying that GluClRs with a greater content of β subunit are less sensitive to IVM. This later inference also suggests that heteromeric αβ GluClRs of *H*. *contortus* can increase the proportion of β subunit as a function of its expression level. This is in contrast to the reported fixed stoichiometry of heteromeric GluClRs of *C*. *elegans* [41].

### IVM analogue potency at α and αβ GluClRs

**Fig 3:**
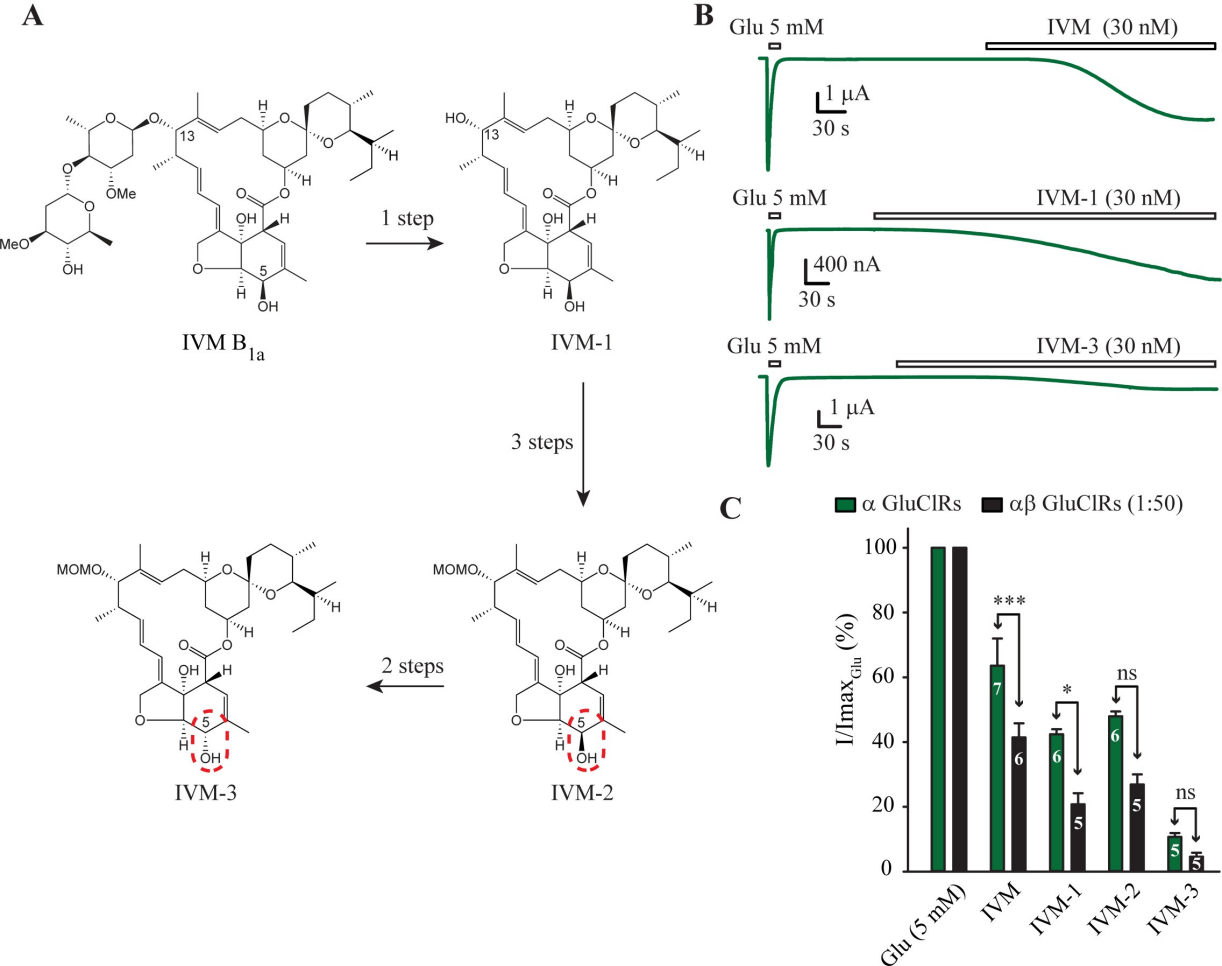
IVM analogue potency. **A.** Structures of IVM and the three analogues. Arrows indicate the synthesis reaction steps. MOMO represents a methoxymethyl ether group. A red broken border indicates the change in 5-OH configuration between IVM-2 and IVM-3. **B.** Example experiments in oocytes expressing α GluClRs that test IVM and analogues, IVM-1 and IVM-3. The IVM or analogue-induced current was normalised to the response elicited by a saturating concentration of glutamate (5 mM). **C.** Group bar plots comparing the potency between α and αβ (1:50) GluClRs for IVM and the three IVM analogues. *** p < 0.001, * p < 0.05. Numbers within the bars indicate the number of experiments (oocytes). Holding potential was −40 mV.

In our next series of experiments, we wished to see if modifying the IVM molecule might alter its potency differentially at α and αβ GluClRs. Three analogues were screened (Fig 3A) at α and αβ GluClRs (1:50) using a standard concentration of 30 nM (Fig 3B). Changes to the IVM parent molecule were made on the basis of IVM and receptor or lipid membrane interactions as revealed by the crystallographic structure of the *C*. *elegans* α GluClR [5].

The maximum current elicited by each IVM analogue was normalised to that induced by a saturating concentration of glutamate (5 mM, Fig 3B) and its efficacy (maximal current) was compared to that of IVM and between α and αβ GluClRs (Fig 3C). The disaccharide moiety is predicted to protrude into the upper leaflet of the cell membrane (Fig 1A) and to form van der Waal (VDW) interactions with the loop that links the M2 and M3 domains of the α subunit of *C*. *elegans* [5] and *H*. *contortus*. Hydrolysis of IVM yielded the aglycone IVM-1 (synthesis details are given in S1 File). IVM-1 retained differential efficacy between the two receptor isoforms but was significantly less efficacious than IVM only at α GluClRs (p < 0.05). Replacing the disaccharide with a methoxymethyl ether group (MOMO) produced IVM-2 (S1 File). This derivative restored efficacy at α but not αβ GluClRs and abolished the differential sensitivity between the receptors. This result suggests that in addition to the predicted M2-M3 loop interactions, steric factors also contribute to the potentiating effects of IVM. IVM-2 (and IVM and IVM-1), with a β-configured 5-OH moiety, is predicted to form VDW interactions and hydrogen bonds with residues of the M2 and M1 domains [5]. The α-configured 5-OH epimer (IVM-3, S1 File), prepared from IVM-2, produced the most marked reduction in potency (p < 0.001 for both GluClRs) relative to IVM. Moreover, this change also ablated the differential efficacy between the two GluClR isoforms (Fig 3C). This result is in accord with the predicted role of the 5–OH moiety, which forms a hydrogen bond with the polar residues within the pore-lining M2 domain (S15’ in *C*. *elegans* α [5] or S16’ and Q15’in *H*. *contortus* α and β, respectively (Fig 1B). A previous study also found this position was important, as subtle changes to 5-O or 5-NOH resulted in dramatic decreases in activity when tested in an *H*. *contortus* larval assay [22].

Overall, differential efficacy between α and αβ GluClRs was maintained for IVM and IVM-1 whereas IVM-2 and IVM-3 were equally efficacious at both receptors. The preservation of efficacy of IVM-1 and IVM-2 compared to IVM at αβ GluClRs suggests that the β subunit likely contributes to IVM binding sites. The disaccharide and the 5-OH moiety of IVM are salient determinants of the potentiating potency of IVM. The data reveal two IVM-receptor interactions that accord well with predictions from structural studies [5] and suggest that with rational drug design it may be possible to develop IVM analogues to selectively target specific GluClR isoforms.

### IPSCs mediated by α and αβ (1:1) GluClRs

Evidence suggesting the existence of glutamate-gated anion-selective post-synaptic receptors was first noted in arthropods over 40 years ago via observing membrane potential changes [2, 3, 42]. However, to our knowledge direct recordings of GluClR-mediated IPSCs have not been made in any invertebrate species. We have recently developed a heteroculture consisting of primary (mammalian) neurons and HEK293 cells transfected with defined post-synaptic receptors of vertebrates, along with the trans-synaptic protein neuroligin [43–45].

**Fig 4:**
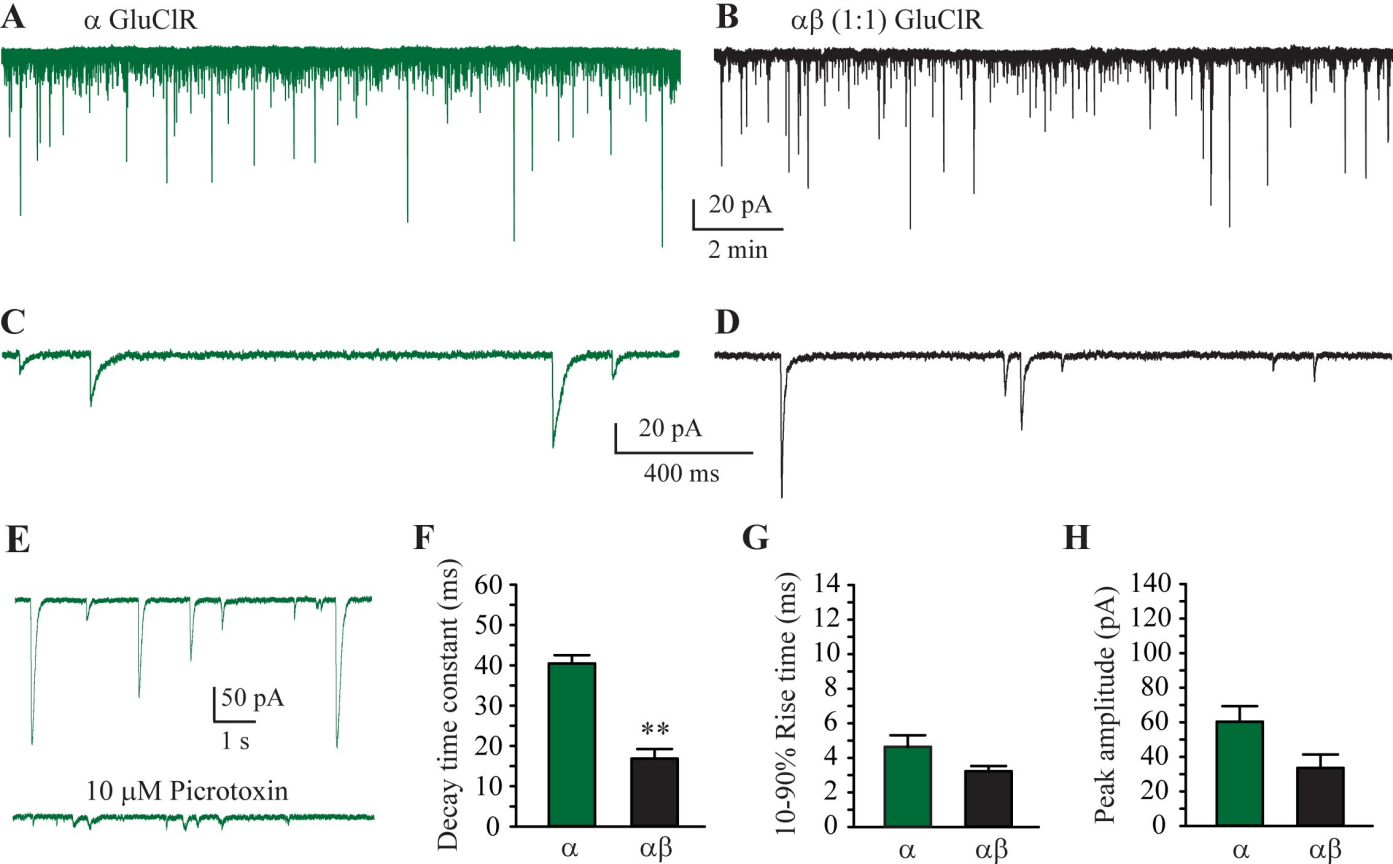
IPSCs mediated by α and αβ GluClRs. (**A-B).** A whole-cell recording from HEK293 cell expressing α GluClRs (**A**) or αβ GluClRs (**B**) in co-culture with primary neurons. **C.** Expanded view of a segment from (A) showing isolated IPSCs that rise rapidly to a peak before decaying back to baseline. **D.** Expanded view of a segment from (B) showing isolated IPSCs that decay faster than those in (C). **E.** Inhibition of IPSCs mediated by α GluClRs by picrotoxin. (**F-H**). Group bar plot showing the mean IPSC decay time constant (F), 10–90% rise times (G) and peak amplitude (H) of α and αβ GluClRs. ** p < 0.01. Holding potential was −70 mV.

This heterosynapse preparation was used to examine if α and αβ GluClRs of *H*. *contortus* cluster at post-synaptic sites and respond to synaptically released glutamate. Cells transfected with the α (Fig 4A) or α and β (1:1, Fig 4B) subunits in co-culture with neurons exhibited prominent currents typical of those expressed at vertebrate synapses, consisting of a fast rise to peak followed by a slower, exponential decay back to baseline (Fig 4C and 4D). These IPSCs were only observed in transfected cells and were blocked by picrotoxin (Fig 4E), confirming that the receptors were anion-selective GluClRs [5, 36]. These data clearly demonstrate that α and αβ GluClRs of *H*. *contortus* generate classic, picrotoxin-sensitive IPSCs. A clear, observable difference between IPSCs mediated by the two receptor isoforms was that αβ GluClRs decayed faster than those mediated by α GluClRs (Fig 4C and 4D). The mean rise-times, peak amplitudes and decay times were measured and plotted as bar plots (Fig 4F–4H). The group averages confirmed that only the decay times between the two receptor isoforms were significantly different, with those of the α GluClRs decaying with a time constant of 40 ± 2 ms (n = 17), whereas the decay time constant for the αβ GluClRs was 17 ± 2 ms (n = 8). The rise times were 4.6 ± 0.7 ms for the α GluClRs and 3.2 ± 0.3 ms for the αβ GluClRs. The peak amplitudes were the most variable of the three parameters, being 60 ± 9 pA and 34 ± 8 pA for the α and αβ GluClRs, respectively, likely reflecting variable expression levels of receptors.

### The effects of IVM at IPSCs of α and αβ (1:1) GluClRs

**Fig 5:**
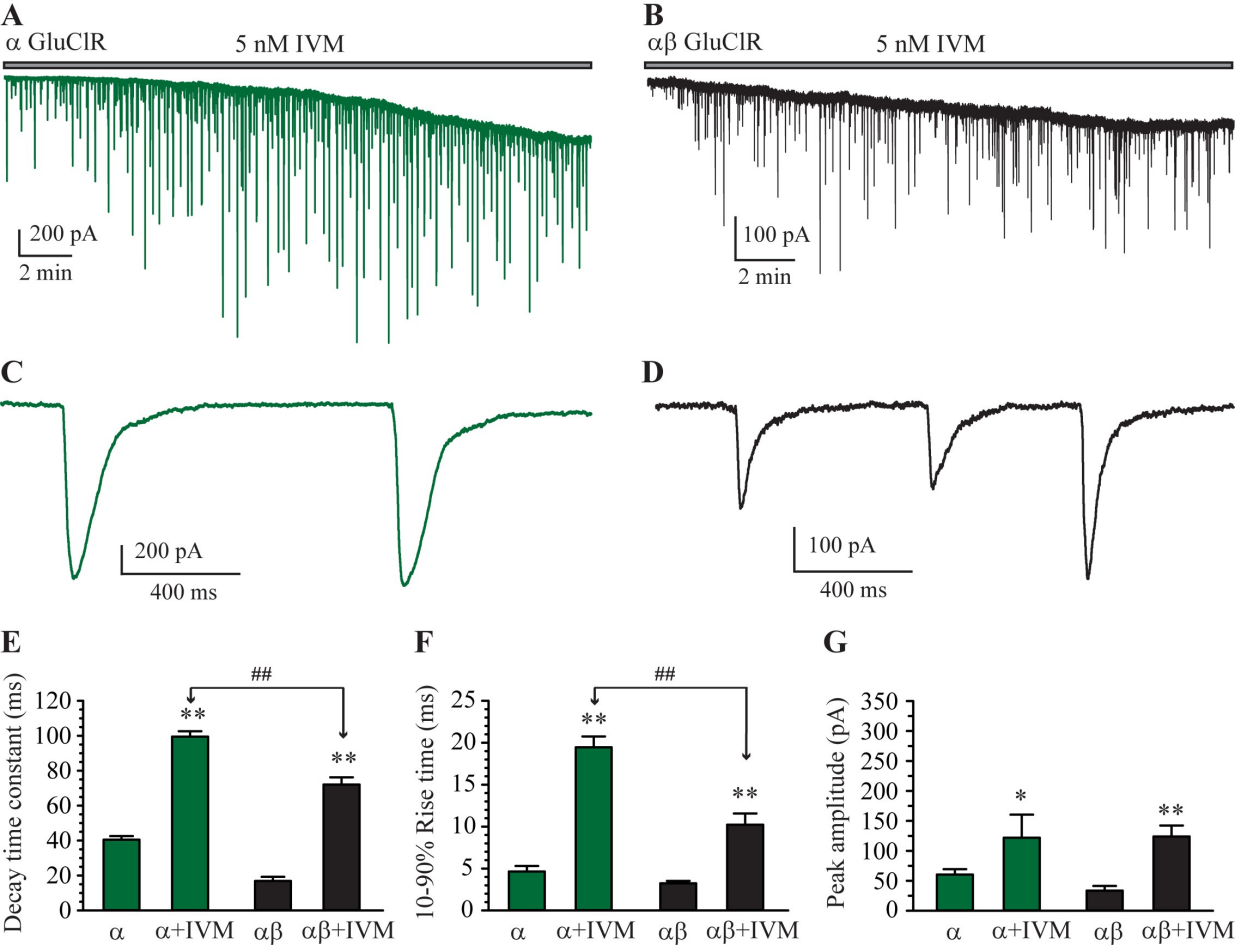
Effects of IVM at IPSCs mediated by α and αβ GluClRs. (**A-B).** A whole-cell recording from a HEK293 cell expressing α GluClRs (A) or αβ GluClRs (B) in co-culture with primary neurons in the continuous presence of 5 nM IVM. Note the steady increase in inward tonic current, which reaches a steady-state plateau at about 17 min in both (A) and (C). **C.** Expanded view of a segment from (A) showing isolated IPSCs, which have larger peak amplitudes and rise and decay more slowly than the corresponding IPSCs in the absence of IVM. **D.** Expanded view of a segment from (B) showing isolated IPSCs, which have larger peak amplitudes and rise and decay more slowly than the corresponding IPSCs in the absence of IVM. Note that the peak amplitude is smaller and the rise and decay times are faster than those in (C). **E-G.** Group bar plot showing the mean IPSC decay time constant (E), 10–90% rise times (F) and peak amplitude (G) of α and αβ GluClRs in the presence and absence of IVM. ** p < 0.01, * p < 0.05, ## p < 0.01. Holding potential was −70 mV.

After validating the heterocultures as a reliable preparation for investigating IPSCs mediated by GluClRs, we made synaptic recordings while continuously applying IVM (5 nM) to cells similarly transfected with α and αβ GluClRs. Two salient effects were observed in these recordings. Firstly, there was a steady downward deflection in baseline current over the course of the recording that was not different between cells expressing α (Fig 5A) and αβ (1:1, Fig 5B) GluClRs. These data were subsequently pooled for analysis of the tonic current component. The tonic currents plateaued at 17 ± 1 minutes (n = 13), had a fitted time constant of 483 ± 72 s and a mean amplitude of 304 ± 54 pA. Changes to the kinetic properties of IPSCs were also observed upon IVM application (Fig 5C–5G). These were analysed 15 minutes after the commencement of the IVM application to ensure that the effects had equilibrated. For both receptor types, the decay and rise times and peak amplitudes increased significantly in the presence of IVM relative to IVM naïve cells. The decay time constant for α GluClRs slowed from 40 ± 2 ms to 100 ± 3 ms (n = 6) and for αβ GluClRs from 17 ± 2 ms to 72 ± 4 ms (n = 6, Fig 5E). The increase in activation times were from 4.6 ± 0.7 ms to 19 ± 1 ms for α GluClRs and from 3.0 ± 0.3 ms to 10 ± 1 ms for the αβ heteromers (Fig 5F). Again, the peak IPSC amplitudes were variable but increased significantly in the presence of IVM from 60 ± 9 pA to 122 ± 39 pA and from 34 ± 8 pA to 124 ± 18 pA for α and αβ GluClRs, respectively (Fig 5G). A comparison of the magnitude changes of IPSC parameters was also made between the two receptor isoforms. Consistently greater decay and rise times were observed for the α GluClRs (Fig 5E and 5F), suggesting that IPSCs mediated by these receptors produce greater inhibitory input than αβ GluClRs in the presence of IVM.

### Single receptor properties of β and αβ (1:1) GluClRs

The decay times of IPSCs mediated by αβ GluClRs were consistently and significantly faster than those of α GluClRs, in both the absence and presence of IVM. We previously showed that the decay times of macropatch currents mediated by α GluClRs incorporating an IVM-insensitive mutation (G36’A) were faster than those mediated by wild-type α GluClRs, and that this was due to briefer single receptor active periods and enhanced receptor desensitisation [35].

**Fig 6:**
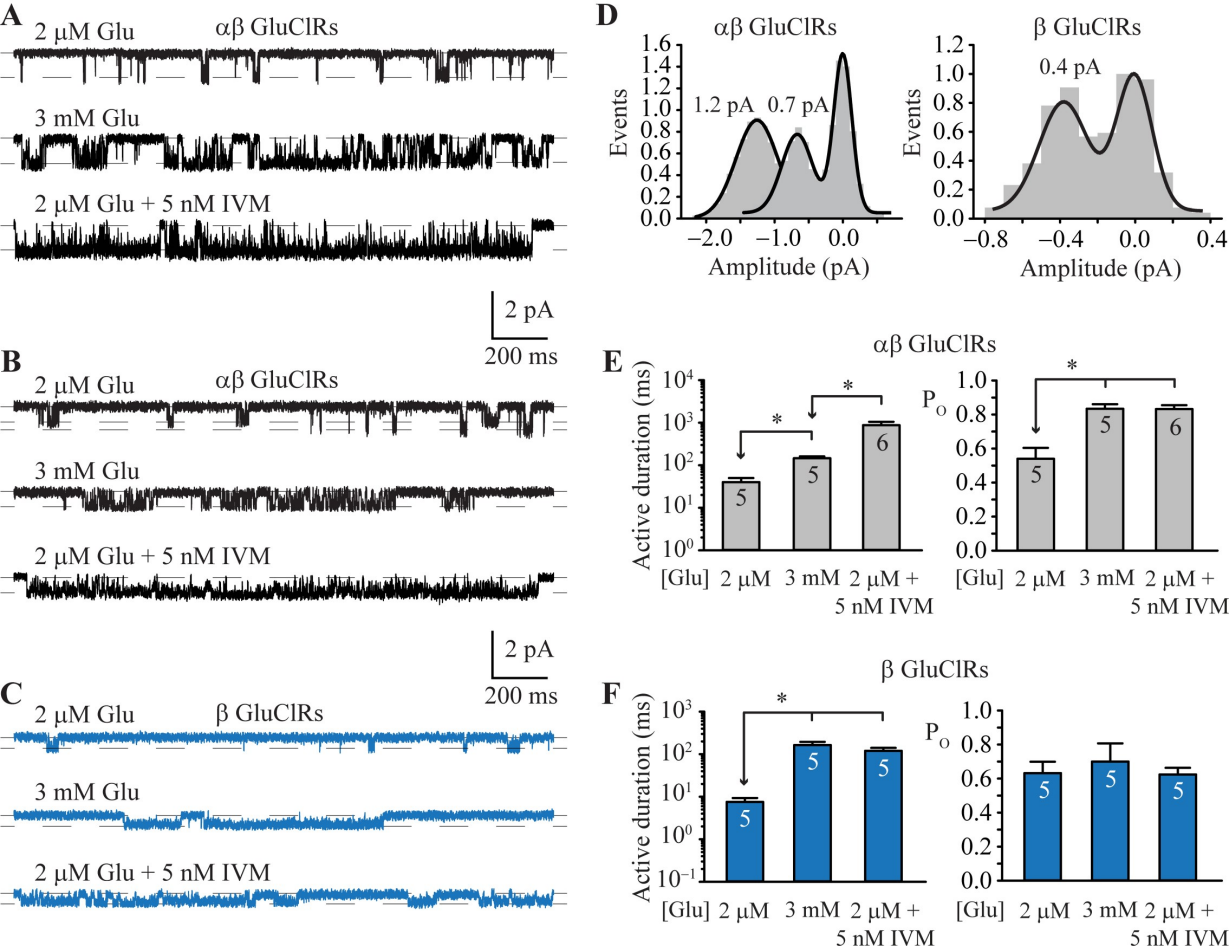
Single channel properties of α and αβ GluClRs. **A.** Single receptor currents from patches expressing αβ (1:1) GluClRs at 2 μM and 3 mM glutamate and 2 μM glutamate plus 5 nM IVM. Note the increase in the duration of the active periods in the presence of IVM. **B.** Transfections of α and β subunits produced two distinct types of single channel activity based on current amplitude (1.2 and 0.7 pA). The 1.2 pA activity was the most prominent, accounting for 90% of the openings. The active durations of both types increase at higher glutamate concentrations and in the presence of IVM. **C.** Single receptors currents mediated by β GluClRs in the presence of the indicated concentrations of glutamate and in 2 μM glutamate plus 5 nM IVM. Note only one amplitude level (0.4 pA). **D.** Amplitude histograms for single receptor activity from patches expressing αβ GluClRs showing the two main amplitude levels (left) and β GluClRs (left). **E.** Single receptor mean active period durations (left) and intra-activation open probability (right) for αβ GluClRs in response to the indicated ligands. **F.** Single receptor mean active period durations (left) and intra-activation open probability (right) for β GluClRs in response to the indicated ligands. * p < 0.05. Numbers within the bars indicate the number of experiments (patches). Holding potential was −70 mV.

To explore whether a similar mechanism applies to β-containing GluClRs, we recorded single receptor currents in excised patches from cells transfected with either the α and β subunits (1:1) or the β subunit alone. These experiments were recorded in 2 μM and 3 mM glutamate as well as 2 μM glutamate plus 5 nM IVM at a clamped potential of ‐70 mV (reversal potential = 4.0 mV, liquid junction potential = 4.7 mV and membrane potential = 78.7 mV) [35]. Patches from cells transfected with both subunits revealed that the majority (~90%) of single αβ receptors opened to an amplitude of 1.2 pA (Fig 6A) with the remainder opening to 0.7 pA (Fig 6B). By contrast, patches from cells transfected with the β subunit alone invariably opened to an amplitude of 0.4 pA (Fig 6C). Amplitude histograms confirmed two amplitude levels for αβ GluClRs and a single amplitude for the β GluClRs (Fig 6D). Our data suggest that a 1:1 transfection ratio of α and β subunits results in a predominant stoichiometry, which exhibits an amplitude of 1.2 pA and a less frequent subunit combination that opens to an amplitude of 0.7 pA. We infer that the αβ GluClRs that open to 0.7 pA likely contain a greater proportion of β subunits. This is consistent with β GluClRs having the smallest amplitude and α GluClRs having the greatest amplitude (1.8 pA) under similar recording conditions [35]. The calculated conductance values for the corresponding amplitudes for αβ GluClRs was 15.2 pS and 8.9 pS and for the β GluClRs was 5.1 pS.

Single receptor active periods were analysed for mean duration and intra-activation open probability (P_O_). For αβ GluClRs the activations from both stoichiometries were pooled to obtain mean active periods of 40 ± 10 ms (n = 5) in 2 μM glutamate and 146 ± 15 ms (n = 5) in 3 mM glutamate. Consistent with the example recordings presented in Fig 6A and 6B the respective P_O_s were 0.54 ± 0.06 and 0.83 ± 0.03 in 2 μM and 3 mM glutamate (Fig 6E). Current potentiation by IVM did not change the proportion of activations to 1.2 pA compared to 0.7 pA, but manifested as a marked increase in the mean duration of the active periods (pooled, 876 ± 178 ms, n = 6) (Fig 6A, 6B and 6E). This represents an over 20-fold increase compared to 2 μM glutamate alone and 6-fold increase compared to 3 mM glutamate. The P_O_ in 2 μM glutamate plus 5 nM IVM was similar to that in 3 mM glutamate, being 0.83 ± 0.02 (Fig 6E).

β GluClRs exhibited the briefest active periods (Fig 6C). At 2 μM glutamate, β GluClRs opened for a mean duration of 7.5 ± 1.8 ms and had a P_O_ of 0.63 ± 0.07 (n = 5) (Fig 6F). The active durations increased in 3 mM glutamate to 164 ± 31 ms, whereas the P_O_ remained unchanged (0.70 ± 0.11, n = 5) (Fig 6F). In contrast to the supersaturating effects IVM has at α and αβ GluClRs, it was ineffective at increasing the duration of active periods of β GluClRs beyond that measured in 3 mM glutamate. The active periods in 2 μM glutamate plus 5 nM IVM were 120 ± 21 ms in duration and the P_O_ was 0.62 ± 0.04 (n = 5) (Fig 6F).

Our single receptor measurements show that by increasing the active durations and P_O_ of single GluClRs, IVM produces an increase in IPSC decay times and peak amplitude, respectively. Our data also demonstrate a correlation between single receptor active duration, IPSC decay times and IVM potentiation of IPSCs.

### Partitioning and diffusion of IVM in cell membranes

**Fig 7:**
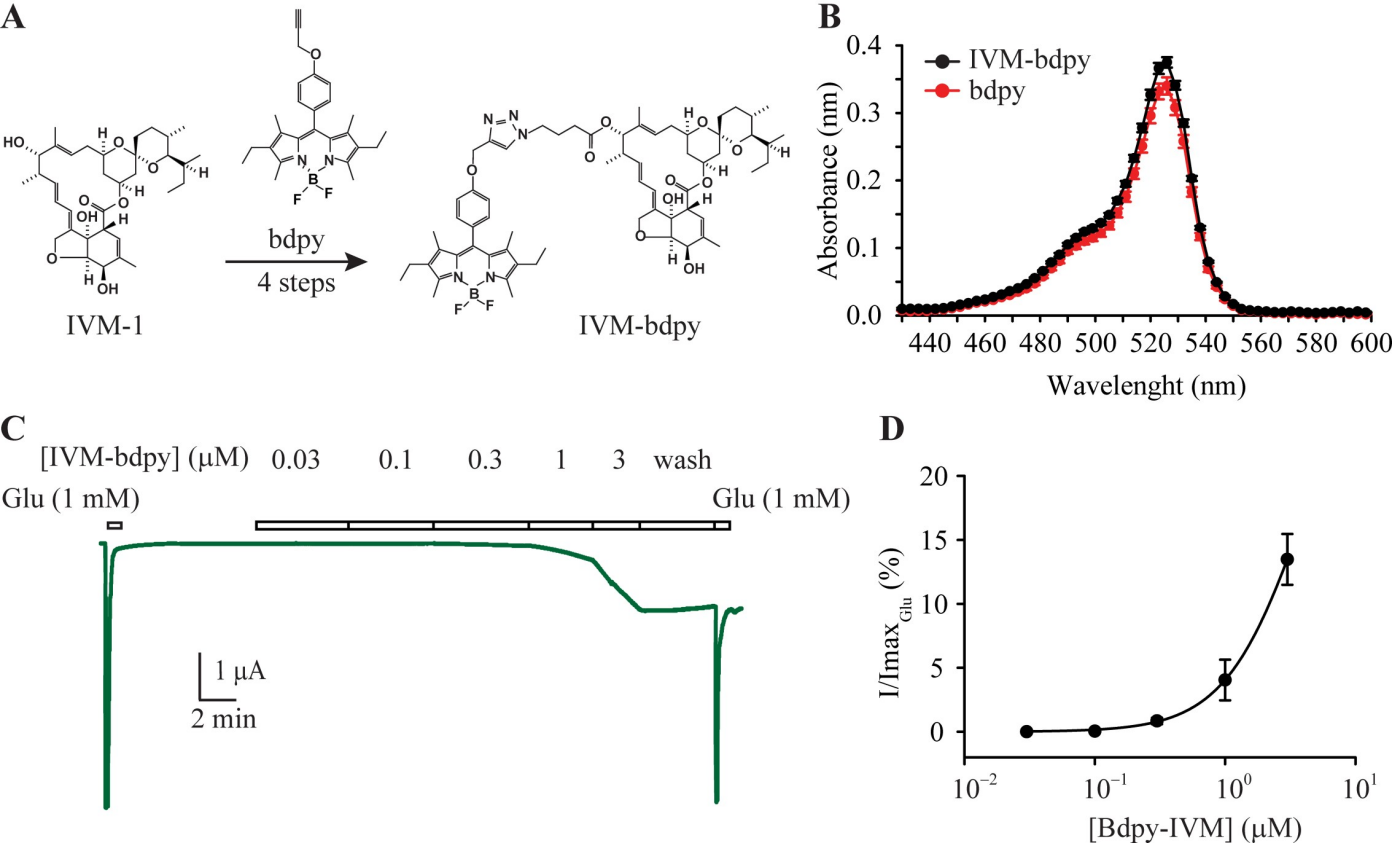
IVM-bdpy fluorescent probe. **A.** Structures of free bdpy fluorophore and IVM-bdpy. **B.** The spectral emission properties of IVM-bdpy and the free bdpy fluorophore. **C.** IVM-bdpy retained activity as shown in the sample oocyte current mediated by α GluClRs. **D.** Group concentration-response plot (n = 6) for IVM-bdpy at α GluClRs, showing reduced potency relative to IVM.

Our IPSC and single channel data suggest that the potentiating effect of IVM at GluClRs requires several minutes to stabilise [35]. This relatively long delay could reflect the slow binding of IVM directly from the aqueous solution or slow IVM-induced conformational effects at GluClRs once bound [46, 47]. Alternatively, it may reflect a slow accumulation rate of IVM into the membrane, possibly coupled with a slow lateral diffusion rate within the membrane that controls its access to receptor binding sites. To help distinguish between these possibilities, we synthesised a fluorescent IVM-bodipy probe (IVM-bdpy, Fig 7A and S1 File) to monitor the interaction of IVM with the cell membrane. This probe consisted of IVM-1, which retains its activity at GluClRs (Fig 3), attached to a bodipy fluorophore. The resulting IVM-bdpy derivative exhibited spectral emission properties similar to free bdpy (Fig 7B) and was active at α GluClRs (Fig 7C), albeit with a reduced potency (Fig 7D).

**Fig 8:**
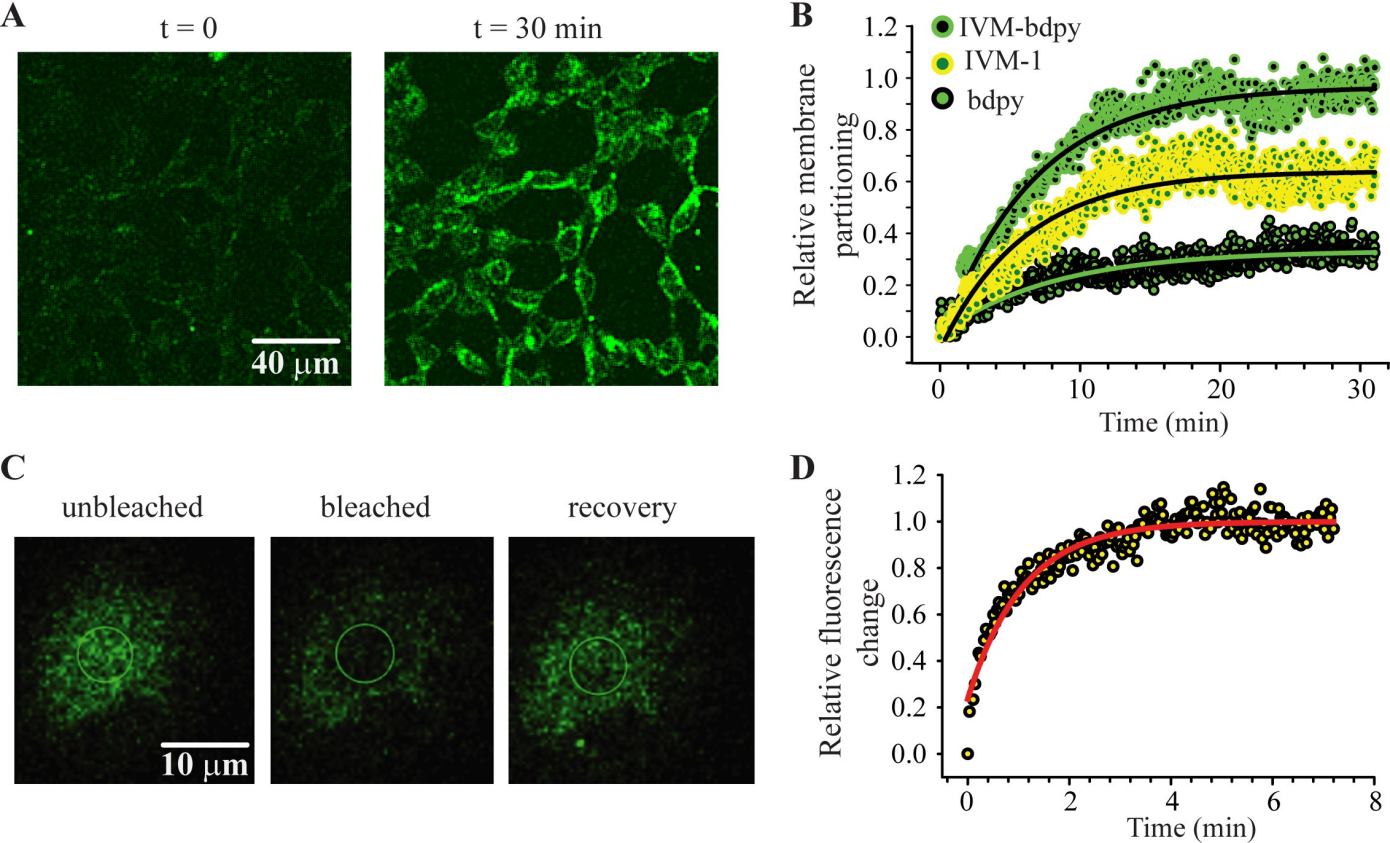
Membrane partitioning and lateral diffusion of IVM-bdpy. **a.** Confocal image of a field of HEK293 cells before (left) and after 30 min exposure of 500 nM IVM-bdpy (right). **B.** Group plot (n = 5) of the change in fluorescence over time for IVM-bdpy (green) the free bdpy fluorophore (black) and the subtraction (IVM-1, yellow). These plots were fitted to exponential functions to yield time constants of 6.5 min for the IVM-bdpy, 8.2 min for the bdpy fluorophore and 6.0 min for IVM-1. **C.** An example of a FRAP experiment showing a cell after equilibrating in 500 nM IVM-bdpy for 30 min (left), after a circular region (demarcated) was pholobleached with laser energy (middle) and after recovery of the bleached region (right). **D.** Group plot (n = 5) of the rate of recovery after pholobleaching. The data were fitted to an exponential with a time constant of 1.3 min.

Two types of experiments were conducted with IVM-bdpy. The first was aimed at monitoring the time-course of membrane accumulation. Untransfected HEK293 cells were incubated in extracellular solution containing 500 nM IVM-bdpy and time-lapse images were taken over a period of 30 min (Fig 8A). The change in total membrane fluorescence averaged from 5 repeat experiments was then plotted against time and fitted to a standard exponential function (Fig 8B). In a control experiment, the corresponding accumulation rate was also determined for bdpy. To obtain the net membrane partitioning time-course for IVM-1, the data for the bdpy alone was subtracted from the data obtained for IVM-bdpy (Fig 8B). The time constants for the three plots were 6.5 min for the IVM-bdpy, 8.2 min for the bdpy fluorophore and 6.0 min for IVM-1. As the lipophilic properties of IVM and IVM-1 are similar (logPs of 5.4 and 5.1, respectively), we infer this analysis quantitatively reflects the membrane partitioning properties of IVM. Notably, the resultant plot plateaued at about 18–20 minutes, which was similar to the estimated time to plateau for the IVM-activated current as presented in Fig 5A and 5B. It should be noted, however, that the IVM-bdpy experiment was done using a 100-fold higher concentration of drug in un-transfected cells and represents passive membrane accumulation, whereas the current plateau involved IVM binding to and activating receptors. Nevertheless, these data suggest that membrane partitioning of IVM is the rate limiting factor controlling the activation rate of GluClRs.

The second experiment was aimed at determining the lateral membrane diffusion properties of IVM using the fluorescence recovery after photobleaching (FRAP) technique. After a 30-min equilibration time, 5 cells were monitored for fluorescence recovery within a bleached circular patch of membrane of 3.7 μm diameter (Fig 8C). Any bleaching and recovery of the surrounding area of the cell was measured, using a similar circular patch and used to correct the recovery rate of the membrane delineated by the bleached area. We also noted that the recovery reached 98 ± 2% of control. The resultant plot, fitted to a standard exponential, is shown in Fig 8D. The time constant (τ) for recovery of 1.3 minutes was used to calculate the diffusion coefficient (D), using the radius (r) of the bleached patch, according to D = r^2^/4τ. The calculated value of D was 1.1 x 10^−2^ μm^2^s^−1^. Over the course of 1 s, the IVM-bdpy probe would traverse 0.2 μm along the membrane surface (lateral diffusion rate of 0.2 μms^−1^). This value is comparable to that calculated using a similar probe that was used to measure partitioning and lateral diffusion in muscle membranes of *Ascaris suum* [39]. We infer that this slow lateral diffusion rate coupled to membrane partitioning are the major contributors to local increases of IVM around its target binding sites and the slow quasi-irreversible actions of IVM at GluClRs.

## Discussion

Resistance to IVM in pest species is a growing problem worldwide. Previous research efforts have identified mutations and allelles of GluClRs associated with resistance to IVM, however, there is a lack of information regarding the mechanisms that underlie resistance. In this study, we investigated the synaptic and biophysical properties of different GluClR subtypes and detailed a possible mode of resistance in the pest species *H*. *contortus*.

We found that β subunits of *H*. *contortus* can assemble both as homomers and as heteromeric combinations with the α subunit. Given that both subunits have common distribution patterns in motor neuron commissures and nerve cords [14], it is possible that these two subunits combine to form heteromeric receptors, which are less sensitive to IVM but retain high sensitivity to neurotransmitter. Our data also support the idea that the subunit stoichiometry of heteromeric GluClRs is variable and that receptors with a greater proportion of β subunits are less sensitive to IVM. With a high EC_50_ (400 μM) for glutamate, a low unitary conductance and brief active periods, homomeric β GluClRs are not likely to be efficacious post-synaptic receptors. Moreover, given that our single channel recordings reveal little homomeric β GluClR activity in patches expressing both α and β subunits, homomeric β GluClRs are likely to be scarce in cells that also express α subunits.

Using heterocultures of neurons and transfected HEK cells, we demonstrate that α and αβ GluClRs mediate IPSCs in response to presynaptic glutamate release. HEK293-neuronal co-cultures have been shown to recapitulate synaptic current profiles in excitatory glutamatergic synapses expressing NMDA or AMPA receptors [48] suggesting that glutamate release in heterosynapses is likely to be physiological. To our knowledge, no published reports are available describing the properties of IPSCs mediated by native GluClRs in invertebrate neurons or muscle cells. Our preparation therefore represents a technological advance with the potential to improve our understanding of (1) the kinetics of IPSCs mediated by defined GluClR isoforms, (2) the effects of drugs on IPSCs mediated by defined GluClR isoforms, (3) the effect of posttranslational modifications (e.g., phosphorylation) and resistance mutations on the formation and function of synapses, and (4) synaptogenesis and synaptic clustering mechanisms. We observed a substantial increase in IPSC peak amplitude and a general slowing of IPSC kinetics, particularly the decay times. IPSC experiments also revealed a tonic inhibitory component, which is a well-studied mode of inhibition in vertebrates that may also involve extrasynaptic pLGICs [49]. In the presence of IVM we demonstrate that whereas the tonic component of inhibition is similar in cells expressing α and αβ GluClRs, the phasic IPSC component is potentiated to a smaller degree in αβ GluClRs. This lower sensitivity is mostly due to the intrinsic activation properties of β-containing receptors. Hence, the net inhibitory signal mediated by αβ GluClRs in the presence of IVM would be less than that of α GluClRs, thereby underlying a possible mechanism of IVM resistance.

Our single channel recordings showed that β-containing GluClRs had briefer active periods and a smaller conductance, both of which render receptors less sensitive to the potentiating effects of IVM when compared to α GluClRs [35].

Single receptor active durations also accord well with ensemble decay times as revealed in macropatches [35] and IPSCs (this study). GluClRs with faster decay times are potentiated to a lesser extent by IVM in absolute terms and thus produce less inhibitory input to target cells. The decay times for the α GluClRs were 67 ms (macropatch) [35] and 40 ms (IPSCs). For GluClRs that exhibited reduced IVM sensitivity the decay times were 11 ms (macropatch) for α(G36’A) [35] and 17 ms (IPSCs) for the αβ GluClRs. It is also noteworthy that vertebrate pLGICs, such as glycine and GABA-gated receptors that are much less sensitive to IVM also exhibit relatively brief single receptor active periods and faster ensemble decay times [43, 50].

We hypothesised that a noteworthy component of the mode of action of IVM was its interactions with cell membranes. A fluorescently tagged IVM (IVM-bdpy) was used to measure the time-course of drug accumulation and lateral diffusion in membranes that was compared to the onset of current potentiation. Single channel currents recorded from small, ~2 μM diameter patches equilibrated over 1–2 minutes [35]. IVM-bdpy required about 18 minutes to saturate whole cell membranes, which was similar to the time taken for baseline currents to plateau (17 minutes) in our IPSC recordings. In addition to the concentrating effects of membrane partitioning, a slow lateral diffusion rate (0.2 μms^−1^) of IVM would also contribute to the apparent association rates of IVM to binding sites at GluClRs [46]. The recovery after bleaching reached near control levels, but was not at 100% for each cell, suggesting the presence of membrane compartments of relatively immobile lipid rafts [51]. Our experiments suggest that the dominant pathway for IVM to reach its binding sites at GluClRs is by membrane partitioning and diffusion rather than inducing slow conformational rearrangements to the receptors after binding directly from the aqueous compartment.

Finally, we sought to identify a new IVM analogue with a differential effect on α and αβ GluClRs as proof of principle that this pharmacophore may be useful in refining anthelminthic treatments by targeting particular GluClR isoforms. We identified key receptor-drug interactions that determine drug potency that include (1) the disaccharide group of IVM and (2) the significance of the C-5 configuration of IVM, which is a key determinant of differential potency between GluClR isoforms.

In summary, we describe the functional properties of α homomeric and αβ heteromeric GluclRs using conventional whole cell recording, single channel analysis and heterosynaptic analysis. Our key findings support the general inference that the intrinsic activation properties of the inhibitory pLGICs is a critical determinant and predictor of the potentiating potency of IVM. Informed by our findings, drug design strategy may be directed towards increasing the active durations of single receptors and slowing decay times of IPSCs in synaptic isoforms of GluClRs.

## Materials and methods

### Molecular biology

cDNAs encoding the α (avr-14b) (pcDNA 3.1+) or β (pUNIV) GluClR subunits of *H*. *contortus* were nuclear injected into oocytes (NASCO, WI USA) or transfected into HEK293AD cells (CellBank Australia) using a calcium phosphate-DNA co-precipitate method. Synapse formation between neurons and HEK293 cells was promoted by co-transfecting neuroligin 2A or 1B and the cDNA encoding CD4 surface antigen was also added to the transfection mixture so as to identify transfected cells. The oocytes and HEK cells were used for experiments 2–3 days after the introduction of the cDNAs. All experiments were done at room temperature (22 ± 1°C).

### Oocyte preparation and two-electrode voltage-clamp

*Xenopus laevis* oocytes were harvested by surgical incision. The oocytes were then defolliculated with 1.5 mg/ml collagenase for 2 hours. Free oocytes were rinsed with calcium-free OR-2 solution containing (in mM) 82.5 NaCl, 2 KCl, 1 MgCl_2_, 5 HEPES, pH 7.4 and mature stage V or VI oocytes were isolated for experiments. The DNAs coding for α and β subunits were nuclear injected into the oocytes using a Nanoliter 2000 microinjector (WPI Inc) at a ratio of (α:β), 1:0, 0:1, 1:1 and 1:50. The total amount of injected DNA in all combinations was 400 ng ml^−1^, including the 1:50 (α:β) combination (8 ng of DNA encoding the α and 392 ng of DNA encoding β subunits). Injected oocytes were incubated in ND96 storage solution (in mM) 96 NaCl, 2 KCl, 1 MgCl2.6H2O, 1.8 CaCl2, 5 HEPES, 50 μg ml^−1^ gentamicin, 2.5 sodium pyruvate, 0.5 theophylline, pH 7.4 at 16°C for 2–3 days before experiment.

For the two-electrode voltage clamp recordings, each oocyte was secured in a cell bath that was continually perfused with ND96 recording solution (ND96 storage solution without pyruvate, theophylline and gentamicin). Glutamate, IVM and IVM analogues were diluted in ND96 recording solution and were applied to the oocyte via bath perfusion. The two microelectrodes contained 3 M KCl and had resistances of 0.2–2 MΩ. Recordings were done using Clampex 10.2 software (Molecular Devices) at a clamped voltage of −40 mV. Currents were low-pass filtered at 200 Hz, sampled at 2 kHz using a Gene Clamp 500B amplifier and digitised by a Digidata 1440A interface. The IVM (IVM B1a) concentration-response experiments were standardised by using an application protocol that consisted of applying increasing concentrations of IVM for, respectively, 3 min, 3 min, 3 min, 2 min, 1 min, 1 min and 0.5 min. The IVM-induced currents were normalised to a saturating glutamate concentration of 5 mM (see example, Fig 2).

### Single channel recordings

Single-channel currents were recorded from outside-out excised patches at a clamped potential of −70 mV. The single channel conductance was calculated by dividing the mean current by the net driving force at a clamped potential of ‐70 mV, after accounting for reversal and liquid junction potentials [35]. Excised patches were continuously perfused via a gravity-fed double-barrelled glass tube, which contained extracellular bath solution containing (in mM), 140 NaCl, 5 KCl, 1 MgCl_2,_ 2 CaCl_2_, 10 HEPES, and 10 D-glucose and titrated to pH 7.4. Glutamate and IVM were dissolved in this extracellular solution. Recording electrodes were pulled from borosilicate glass (G150F-3; Warner Instruments), coated with a silicone elastomer (Sylgard-184; Dow Corning) and heat-polished to a final tip resistance of 4–15 MΩ when filled with an intracellular solution containing (in mM) 145 CsCl, 2 MgCl_2_, 2 CaCl_2_, 10 HEPES, and 5 EGTA, pH 7.4. Stock solutions of L-glutamate were also pH-adjusted to 7.4 with NaOH. A 10 mM stock of IVM (Sigma-Aldrich) was dissolved in 100% DMSO and kept frozen at ‐20°C. Fresh working stocks of IVM at 5 nM were prepared by dissolving the appropriate quantity directly in extracellular solution. 100% DMSO when dissolved in extracellular solution alone at the same concentration as was present in working solutions containing 5 nM IVM had no effect on patches excised from cells transfected with GluClRs or from untransfected cells.

Currents were recorded using an Axopatch 200B amplifier (Molecular Devices), filtered at 5 kHz and digitized at 20 kHz using Clampex (pClamp 10 suite, Molecular Devices) via a Digidata 1440A digitizer.

### Heteroculture preparation and IPSC recording

Primary neuronal cultures were prepared from cortices of E18 rat embryos, (University of Queensland Biological Services), which were triturated and plated at 100,000 cells per 18-mm poly-D-lysine-coated coverslip in DMEM with 10% fetal bovine serum. After 24 h the entire medium was replaced with Neurobasal medium that included 2% B27 and 1% GlutaMAX supplements. After one week half of this medium was replaced and the neurons were allowed to grow *in vitro* for 3–5 weeks before introducing the transfected HEK293 cells. Recordings of synaptic currents were done in whole-cell configuration at ‐70 mV using an Axopatch 200B amplifier (Molecular Devices), filtered at 5 kHz and digitized at 20 kHz using Clampex (pClamp 10 suite, Molecular Devices) via a Digidata 1440A digitizer.

### Microscopy

Membrane partitioning and FRAP experiments were performed on an LSM 710 inverted 2-photon confocal microscope equipped with a 40X water immersion objective lens (1.2 NA/280 μM WD/0.208 μM/pixel and a GFP/Alexa 488 filter set. Untransfected HEK293 cells at a confluency of 50–75% were plated onto 35-mm glass bottom dishes one day prior to experiments. The IVM-bdpy fluorophore was dissolved in 100% DMSO and stored at ‐20°C at a concentration of 24 mM.

A Mai Tai eHP 2-photon laser/760-1040 nm was used as an excitation source (power at 920 nm) for the FRAP experiments. The laser and bleaching power were set to 7% and 75% respectively. Membrane partitioning images were taken for 35 min at 0.5 Hz after replacing the cell medium (extracellular solution) with one containing the IVM-bdpy probe at a concentration of 500 nM. FRAP images were taken at a frequency of 0.5 Hz for 10 min after a 30 min equilibration period in 500 nM IVM-bdpy.

### Analysis and statistics

Group data were analysed in SigmaPlot 13.0 using one-way ANOVAs, where p < 0.05 was taken as the significance threshold and expressed as mean ± SEM. Tests for normally distributed data are built into the SigmaPlot software. Confocal images were captured and analysed with Zen 2012 SP2 (Zeiss) software. Oocyte concentration-response data were fit to a Hill equation to obtain an EC_50_ and Hill co-efficient for each oocyte recording. These parameters were then averaged across multiple oocyte experiments of the same type. Single channel recordings were analysed in QuB software. Single channel currents were idealised and separated into discrete activations by applying critical shut times of 50 ms in glutamate alone or 50–100 ms in glutamate plus IVM to separate and define single receptor active periods. Patches yielded between 30–250 individual active periods. Critical shut times were determined by generating an initial shut duration histogram to continuous data that included inactive periods corresponding to receptor desensitisation. The selected critical times eliminated periods of receptor desensitisation while retaining singe receptor active periods. Mean active period duration and intra-activation open probability was estimated from each patch. Group means were obtained by averaging across multiple patches for each recording condition.

## Supporting information

S1 File Chemical synthesis details of ivermectin analogues, IVM-1, IVM-2, IVM-3 and IVM-bdpy. (DOCX)

## References

[1] Cully DF, Vassilatis DK, Liu KK, Paress PS, Van der Ploeg LH, Schaeffer JM, et al Cloning of an avermectin-sensitive glutamate-gated chloride channel from Caenorhabditis elegans. Nature. 1994;371(6499):707–11. Epub 1994/10/20. 10.1038/371707a0 . 7935817

[2] Marder E, Paupardin-Tritsch D. The pharmacological properties of some crustacean neuronal acetylcholine, gamma-aminobutyric acid, and L-glutamate responses. J Physiol. 1978;280:213–36. 211227 10.1113/jphysiol.1978.sp012381 PMC1282656

[3] Marder E, Eisen JS. Electrically coupled pacemaker neurons respond differently to same physiological inputs and neurotransmitters. J Neurophysiol. 1984;51(6):1362–74. Epub 1984/06/01. 10.1152/jn.1984.51.6.1362 . 6145758

[4] Althoff T, Hibbs RE, Banerjee S, Gouaux E. X-ray structures of GluCl in apo states reveal a gating mechanism of Cys-loop receptors. Nature. 2014;512(7514):333–7. Epub 2014/08/22. 10.1038/nature13669 25143115 PMC4255919

[5] Hibbs RE, Gouaux E. Principles of activation and permeation in an anion-selective Cys-loop receptor. Nature. 2011;474(7349):54–60. Epub 2011/05/17. 10.1038/nature10139 21572436 PMC3160419

[6] Lynagh T, Lynch JW. A glycine residue essential for high ivermectin sensitivity in Cys-loop ion channel receptors. Int J Parasitol. 2010;40(13):1477–81. Epub 2010/08/18. 10.1016/j.ijpara.2010.07.010 . 20713056

[7] Callau-Vazquez D, Pless SA, Lynagh T. Investigation of Agonist Recognition and Channel Properties in a Flatworm Glutamate-Gated Chloride Channel. Biochemistry. 2018;57(8):1360–8. Epub 2018/02/08. 10.1021/acs.biochem.7b01245 . 29411605

[8] Paini DR, Sheppard AW, Cook DC, De Barro PJ, Worner SP, Thomas MB. Global threat to agriculture from invasive species. Proc Natl Acad Sci U S A. 2016;113(27):7575–9. 10.1073/pnas.1602205113 27325781 PMC4941431

[9] Fitzpatrick JL. Global food security: the impact of veterinary parasites and parasitologists. Vet Parasitol. 2013;195(3–4):233–48. 10.1016/j.vetpar.2013.04.005 . 23622818

[10] Tigchelaar M, Battisti DS, Naylor RL, Ray DK. Future warming increases probability of globally synchronized maize production shocks. Proc Natl Acad Sci U S A. 2018;115(26):6644–9. Epub 2018/06/13. 10.1073/pnas.1718031115 29891651 PMC6042138

[11] Horsberg TE. Avermectin use in aquaculture. Current pharmaceutical biotechnology. 2012;13(6):1095–102. Epub 2011/11/02. . 22039799 10.2174/138920112800399158

[12] Wolstenholme AJ. Glutamate-gated chloride channels. The Journal of biological chemistry. 2012;287(48):40232–8. Epub 2012/10/06. 10.1074/jbc.R112.406280 23038250 PMC3504739

[13] Laing R, Gillan V, Devaney E. Ivermectin—Old Drug, New Tricks? Trends in parasitology. 2017;33(6):463–72. Epub 2017/03/14. 10.1016/j.pt.2017.02.004 28285851 PMC5446326

[14] Portillo V, Jagannathan S, Wolstenholme AJ. Distribution of glutamate-gated chloride channel subunits in the parasitic nematode Haemonchus contortus. J Comp Neurol. 2003;462(2):213–22. 10.1002/cne.10735 . 12794744

[15] Wolstenholme AJ, Rogers AT. Glutamate-gated chloride channels and the mode of action of the avermectin/milbemycin anthelmintics. Parasitology. 2005;131 Suppl:S85–95. Epub 2006/03/30. 10.1017/S0031182005008218 . 16569295

[16] Geary TG, Sims SM, Thomas EM, Vanover L, Davis JP, Winterrowd CA, et al Haemonchus contortus: ivermectin-induced paralysis of the pharynx. Exp Parasitol. 1993;77(1):88–96. 10.1006/expr.1993.1064 . 8344410

[17] Wolstenholme AJ, Maclean MJ, Coates R, McCoy CJ, Reaves BJ. How do the macrocyclic lactones kill filarial nematode larvae? Invert Neurosci. 2016;16(3):7 Epub 2016/06/10. 10.1007/s10158-016-0190-7 27279086 PMC5472989

[18] Oerke EC. Crop losses to pests. Journal of Agricultural Science. 2005;144(1):31–43. 10.1017/S0021859605005708

[19] Osei-Atweneboana MY, Awadzi K, Attah SK, Boakye DA, Gyapong JO, Prichard RK. Phenotypic evidence of emerging ivermectin resistance in Onchocerca volvulus. PLoS Negl Trop Dis. 2011;5(3):e998 10.1371/journal.pntd.0000998 21468315 PMC3066159

[20] Kaplan RM, Vidyashankar AN. An inconvenient truth: global worming and anthelmintic resistance. Vet Parasitol. 2012;186(1–2):70–8. Epub 2011/12/14. 10.1016/j.vetpar.2011.11.048 . 22154968

[21] Bebber DP, Ramotowski MAT, Gurr SJ. Crop pests and pathogens move polewards in a warming world. Nature Climate Change. 2013;3:985 10.1038/nclimate1990 https://www.nature.com/articles/nclimate1990#supplementary-information.

[22] Michael B, Meinke PT, Shoop W. Comparison of ivermectin, doramectin, selamectin, and eleven intermediates in a nematode larval development assay. J Parasitol. 2001;87(3):692–6. Epub 2001/06/28. 10.1645/0022-3395(2001)087[0692:COIDSA]2.0.CO;2 . 11426737

[23] Wang X, Puinean AM, AO OR, Williamson MS, Smelt CLC, Millar NS, et al Mutations on M3 helix of Plutella xylostella glutamate-gated chloride channel confer unequal resistance to abamectin by two different mechanisms. Insect Biochem Mol Biol. 2017;86:50–7. 10.1016/j.ibmb.2017.05.006 . 28576654

[24] Wang X, Wang R, Yang Y, Wu S, O'Reilly AO, Wu Y. A point mutation in the glutamate-gated chloride channel of Plutella xylostella is associated with resistance to abamectin. Insect Mol Biol. 2016;25(2):116–25. Epub 2015/11/26. 10.1111/imb.12204 . 26592158

[25] Dermauw W, Ilias A, Riga M, Tsagkarakou A, Grbic M, Tirry L, et al The cys-loop ligand-gated ion channel gene family of Tetranychus urticae: implications for acaricide toxicology and a novel mutation associated with abamectin resistance. Insect Biochem Mol Biol. 2012;42(7):455–65. 10.1016/j.ibmb.2012.03.002 . 22465149

[26] Kwon DH, Yoon KS, Clark JM, Lee SH. A point mutation in a glutamate-gated chloride channel confers abamectin resistance in the two-spotted spider mite, Tetranychus urticae Koch. Insect Mol Biol. 2010;19(4):583–91. 10.1111/j.1365-2583.2010.01017.x . 20522121

[27] Mermans C, Dermauw W, Geibel S, Van Leeuwen T. A G326E substitution in the glutamate-gated chloride channel 3 (GluCl3) of the two-spotted spider mite Tetranychus urticae abolishes the agonistic activity of macrocyclic lactones. Pest Manag Sci. 2017 10.1002/ps.4677 . 28736919

[28] Kane NS, Hirschberg B, Qian S, Hunt D, Thomas B, Brochu R, et al Drug-resistant Drosophila indicate glutamate-gated chloride channels are targets for the antiparasitics nodulisporic acid and ivermectin. Proc Natl Acad Sci U S A. 2000;97(25):13949–54. Epub 2000/11/30. 10.1073/pnas.240464697 11095718 PMC17681

[29] Amanzougaghene N, Fenollar F, Diatta G, Sokhna C, Raoult D, Mediannikov O. Mutations in GluCl associated with ivermectin Field-Resistant Head lice from Senegal. Int J Antimicrob Agents. 2018 Epub 2018/07/29. 10.1016/j.ijantimicag.2018.07.005 . 30055248

[30] Ghosh R, Andersen EC, Shapiro JA, Gerke JP, Kruglyak L. Natural variation in a chloride channel subunit confers avermectin resistance in C. elegans. Science. 2012;335(6068):574–8. Epub 2012/02/04. 10.1126/science.1214318 22301316 PMC3273849

[31] El-Abdellati A, De Graef J, Van Zeveren A, Donnan A, Skuce P, Walsh T, et al Altered avr-14B gene transcription patterns in ivermectin-resistant isolates of the cattle parasites, Cooperia oncophora and Ostertagia ostertagi. Int J Parasitol. 2011;41(9):951–7. Epub 2011/06/21. 10.1016/j.ijpara.2011.04.003 . 21683704

[32] Laing R, Maitland K, Lecova L, Skuce PJ, Tait A, Devaney E. Analysis of putative resistance gene loci in UK field populations of Haemonchus contortus after 6years of macrocyclic lactone use. Int J Parasitol. 2016;46(10):621–30. Epub 2016/05/18. 10.1016/j.ijpara.2016.03.010 27179994 PMC5011429

[33] Rose H, Hoar B, Kutz SJ, Morgan ER. Exploiting parallels between livestock and wildlife: Predicting the impact of climate change on gastrointestinal nematodes in ruminants. Int J Parasitol Parasites Wildl. 2014;3(2):209–19. Epub 2014/09/10. 10.1016/j.ijppaw.2014.01.001 25197625 PMC4152262

[34] Williamson SM, Storey B, Howell S, Harper KM, Kaplan RM, Wolstenholme AJ. Candidate anthelmintic resistance-associated gene expression and sequence polymorphisms in a triple-resistant field isolate of Haemonchus contortus. Mol Biochem Parasitol. 2011;180(2):99–105. Epub 2011/09/29. 10.1016/j.molbiopara.2011.09.003 . 21945142

[35] Atif M, Estrada-Mondragon A, Nguyen B, Lynch JW, Keramidas A. Effects of glutamate and ivermectin on single glutamate-gated chloride channels of the parasitic nematode H. contortus. PLoS pathogens. 2017;13(10):e1006663 10.1371/journal.ppat.1006663 . 28968469 PMC5638611

[36] McCavera S, Rogers AT, Yates DM, Woods DJ, Wolstenholme AJ. An ivermectin-sensitive glutamate-gated chloride channel from the parasitic nematode Haemonchus contortus. Mol Pharmacol. 2009;75(6):1347–55. Epub 2009/04/02. 10.1124/mol.108.053363 19336526 PMC2684884

[37] Lynagh T, Lynch JW. Molecular mechanisms of Cys-loop ion channel receptor modulation by ivermectin. Front Mol Neurosci. 2012;5:60 Epub 2012/05/16. 10.3389/fnmol.2012.00060 22586367 PMC3345530

[38] Alvarez LI, Mottier ML, Lanusse CE. Drug transfer into target helminth parasites. Trends in parasitology. 2007;23(3):97–104. Epub 2007/01/24. 10.1016/j.pt.2007.01.003 . 17236810

[39] Martin RJ, Kusel JR. On the distribution of a fluorescent ivermectin probe (4" 5,7 dimethyl-bodipy proprionylivermectin) in Ascaris membranes. Parasitology. 1992;104 (Pt 3):549–55. . 1641253 10.1017/s0031182000063812

[40] Hartiadi LY, Ahring PK, Chebib M, Absalom NL. High and low GABA sensitivity alpha4beta2delta GABAA receptors are expressed in Xenopus laevis oocytes with divergent stoichiometries. Biochem Pharmacol. 2016;103:98–108. Epub 2016/01/18. 10.1016/j.bcp.2015.12.021 . 26774457

[41] Degani-Katzav N, Gortler R, Gorodetzki L, Paas Y. Subunit stoichiometry and arrangement in a heteromeric glutamate-gated chloride channel. Proc Natl Acad Sci U S A. 2016 10.1073/pnas.1423753113 . 26792524 PMC4747773

[42] Cull-Candy SG. Two types of extrajunctional L-glutamate receptors in locust muscle fibres. J Physiol. 1976;255(2):449–64. Epub 1976/02/01. 1255528 10.1113/jphysiol.1976.sp011289 PMC1309257

[43] Dixon C, Sah P, Lynch JW, Keramidas A. GABAA receptor alpha and gamma subunits shape synaptic currents via different mechanisms. The Journal of biological chemistry. 2014;289(9):5399–411. Epub 2014/01/16. 10.1074/jbc.M113.514695 24425869 PMC3937617

[44] Chen X, Keramidas A, Lynch JW. Physiological and pharmacological properties of inhibitory postsynaptic currents mediated by alpha5beta1gamma2, alpha5beta2gamma2 and alpha5beta3gamma2 GABAA receptors. Neuropharmacology. 2017;125:243–53. 10.1016/j.neuropharm.2017.07.027 . 28757051

[45] Zhang Y, Dixon CL, Keramidas A, Lynch JW. Functional reconstitution of glycinergic synapses incorporating defined glycine receptor subunit combinations. Neuropharmacology. 2015;89:391–7. 10.1016/j.neuropharm.2014.10.026 . 25445488

[46] Sykes DA, Parry C, Reilly J, Wright P, Fairhurst RA, Charlton SJ. Observed drug-receptor association rates are governed by membrane affinity: the importance of establishing "micro-pharmacokinetic/pharmacodynamic relationships" at the beta2-adrenoceptor. Mol Pharmacol. 2014;85(4):608–17. Epub 2014/01/31. 10.1124/mol.113.090209 . 24476583

[47] Degani-Katzav N, Klein M, Har-Even M, Gortler R, Tobi R, Paas Y. Trapping of ivermectin by a pentameric ligand-gated ion channel upon open-to-closed isomerization. Sci Rep. 2017;7:42481 10.1038/srep42481 28218274 PMC5317004

[48] Fu Z, Washbourne P, Ortinski P, Vicini S. Functional excitatory synapses in HEK293 cells expressing neuroligin and glutamate receptors. J Neurophysiol. 2003;90(6):3950–7. Epub 2003/08/22. 10.1152/jn.00647.2003 . 12930820

[49] Jia F, Yue M, Chandra D, Keramidas A, Goldstein PA, Homanics GE, et al Taurine is a potent activator of extrasynaptic GABA(A) receptors in the thalamus. J Neurosci. 2008;28(1):106–15. Epub 2008/01/04. 10.1523/JNEUROSCI.3996-07.2008 . 18171928 PMC6671153

[50] Scott S, Lynch JW, Keramidas A. Correlating structural and energetic changes in glycine receptor activation. The Journal of biological chemistry. 2015;290(9):5621–34. Epub 2015/01/13. 10.1074/jbc.M114.616573 25572390 PMC4342475

[51] Lingwood D, Simons K. Lipid rafts as a membrane-organizing principle. Science. 2010;327(5961):46–50. Epub 2010/01/02. 10.1126/science.1174621 . 20044567

