# Supplementary material for: GluClR-mediated inhibitory postsynaptic currents reveal targets for ivermectin and potential mechanisms of ivermectin resistance
Source: PLoS Pathog. 2019 Jan 29;15(1):e1007570. doi: 10.1371/journal.ppat.1007570 (PMC6368337; doi:10.1371/journal.ppat.1007570)
Supplement: S1 File — (DOCX) [file ppat.1007570.s001.docx]

**Supporting Information**

GluClR-mediated inhibitory postsynaptic currents reveal targets for ivermectin and potential mechanisms of ivermectin resistance

Atif M, Smith JJ, Estrada-Mondragon A, Xiao X, Salim AA,Capon RJ, Lynch JW and

Keramidas A

Correspondence can be sent to Joseph Lynch or Angelo Keramidas

Email: [j.lynch@uq.edu.au](mailto:j.lynch@uq.edu.au) or [a.keramidas@uq.edu.au](mailto:a.keramidas@uq.edu.au)

This PDF file includes:

Chemical synthesis of ivermectin analogues, IVM-1, IVM-2, IVM-3 and IVM-bdpy

**General Experimental Detail for Chemical Synthesis**

NMR experiments were performed on a Bruker Avance DRX600 spectrometer and referenced to residual ^1^H signals in the deuterated solvents. ESIMS experiments were carried out on an Agilent 1100 series LC/MSD instrument. HR-ESIMS data were acquired on a Bruker micrOTOF mass spectrometer by direct infusion in MeCN at 3 μL/min using sodium formate clusters as an internal calibrant. All HPLC analyses and purifications were performed on Agilent 1100 series LC instruments with corresponding detectors, collectors and software inclusively. All chemicals were purchased from Merck, Sigma-Aldrich or Fluka. Solvents used for general purposes were of at least analytical grade, and solvents used for HPLC were of HPLC grade. Phenomenex Luna C8, 10 µm, 210 × 250 mm column (preparative) columns were used for HPLC separations. Flash column chromatography was performed on silica gel LC60A (40–63 mm, Davisil).

**Scheme S1.** Synthetic scheme for IVM analogs, bdpy and IVM-bdpy. Reagents and conditions: (a) 2% H_2_SO_4_, MeOH, rt, 16 h, (b) DMAP, imidazole, TBDMSCl, DCM, rt, 16 h, (c) MOMCl, DIPEA, DCM, rt, 16 h, (d) HF.pyridine, THF, rt, 16 h, (e) DIAD, PPh_3_, HCOOH, THF, rt, 16 h, (f) NaHCO_3_, MeOH/THF/H_2_O, rt, 16 h, (g) (1) NaN_3_, Acetone/H_2_O, reflux, 16 h, (2) KOH, MeOH/H_2_O, rt, 16 h, (h) (COCl)_2_, cat. DMF, DCM, rt, 3 h, (i) TEA/DMAP, DCM, rt, 16 h, (j) 1% TsOH/MeOH, rt, 16 h, (k) (1) TFA, DCM, rt, (2) DDQ, rt, (3) Et_3_N/BF3, Et_2_O, rt, (l) CuSO_4_.5H_2_O, sodium ascorbate, CH_3_CN, 82 °C, 5 d.

**Preparation of IVM-1**

To a solution of **IVM** (100 g, 0.11 moL) in MeOH (1 L) was added concentrated sulfuric acid (20 mL) dropwise, and the reaction was stirred overnight. Thin layer chromatography (TLC) was utilised to monitor the reaction. After removal of the solvent *in vacuo*, the residue was poured into iced water and quenched with Na_2_CO_3_. The mixture was extracted with EtOAc (3 × 300 mL). The combined organic layers were washed with brine, dried over anhydrous MgSO_4_, filtered, concentrated *in vacuo*, and purified with silica gel chromatography (EtOAc/hexane = 1:1) to yield **IVM-1** (51 g, 76%). ^1^H NMR (600 MHz, CDCl_3_), see Figure S3: δ_H_ 5.83 (d, *J* = 12 Hz, 1H), 5.70 (m, 1H), 5.69 (m, 1H), 5.41 (s, 1H), 5.30 (m, 1H), 5.29 (m, 1H), 4.70 (dd, *J* = 14.0, *J* = 2.0 Hz, 1H), 4.66 (dd, *J* = 14.0, *J* = 2.0 Hz, 1H), 4.32 (m, 1H), 4.01 (br, 1H), 3.97 (d, *J* = 6 Hz, 1H), 3.67 (m, 1H), 3.25 (m, 1H), 3.20 (m, 1H), 2.53 (m, 1H), 2.30 (m, 2H), 1.98 (m, 1H), 1.86 (s, 3H), 1.75 (m, 1H), 1.65 (m, 1H), 1.51 (m, 1H), 1.50 (s, 3H), 1.50 (m, 2H), 1.49 (m, 1H), 1.48 (m, 1H), 1.42 (m, 2H), 1.33 (m, 1H), 1.17 (d, *J* = 7.2 Hz, 3H), 0.96 (t, *J* = 7.2 Hz, 3H), 0.85 (d, *J* = 6.6 Hz, 3H), 0.81 (m, 1H), 0.79 (d, *J* = 6.6 Hz, 3H); ^13^C NMR (150 MHz, CDCl_3_), see Figure S4: δ_C_ 173.4, 139.7, 138.6, 137.6, 137.0, 124.7, 120.4, 118.1, 117.1, 97.4, 80.2, 79.2, 77.5, 77.2, 68.5, 68.4, 67.7, 67.3, 45.6, 41.3, 40.0, 36.7, 35.7, 35.5, 34.2, 31.2, 28.0, 27.4, 19.9, 19.1, 17.4, 14.6, 12.5, 11.7. HRESIMS *m*/*z* 609.3399 [M + Na] ^+^ (calcd for C_34_H_50_O_8_Na, 609.3398), see Figure S5.

**Preparation of IVM-2**

To a solution of **IVM-1** (47.0 g, 0.08 moL), 4-dimethylaminopyridine (DMAP, 0.97 g, 0.008 moL) and imidazole (54.5 g, 0.8 moL) in DCM (1 L) at 0 °C was added tert-butyldimethylsilyl chloride (TBDMSCl, 26.6 g, 0.176 moL), and the reaction was allowed to reach ambient temperature and stirred overnight. TLC was utilised to monitor the reaction. After washing with water (300 mL) and brine (300 mL), the organic layer was dried over anhydrous MgSO_4_, filtered, concentrated *in vacuo*, and purified with silica gel chromatography (EtOAc/hexane = 1:5), to yield (**i**) (47.7 g, 85%). ^1^H NMR (600 MHz, CDCl_3_): δ_H_ 5.77 (d, *J* = 12.0 Hz, 1H), 5.71 (m, 1H), 5.69 (m, 1H), 5.32 (m, 1H), 5.32 (m, 1H), 5.26 (m, 1H), 4.66 (dd, *J* = 14.0, *J* = 2.0 Hz, 1H), 4.56 (dd, *J* = 14.0, *J* = 2.0 Hz, 1H), 4.42 (br, 1H), 3.99 (br, 1H), 3.80 (d, *J* = 6.0 Hz, 1H), 3.67 (m, 1H), 3.34 (m, 1H), 3.18 (m, 1H), 2.51 (m, 1H), 2.30 (m, 2H), 1.98 (m, 1H), 1.78 (s, 3H), 1.73 (m, 1H), 1.64 (m, 1H), 1.52 (m, 1H), 1.52 (s, 3H), 1.50 (m, 2H), 1.49 (m, 1H), 1.47 (m, 1H), 1.41 (m, 2H), 1.31 (m, 1H), 1.16 (d, *J* = 7.2 Hz, 3H), 0.94 (t, *J* = 7.2 Hz, 3H), 0.91 (s, 9H), 0.83 (d, *J* = 6.6 Hz, 3H), 0.82 (m, 1H), 0.78 (d, *J* = 6.6 Hz, 3H), 0.12 (s, 6H); ^13^C NMR (150 MHz, CDCl_3_): δ_C_ 173.7, 140.3, 138.7, 137.3, 136.5, 124.8, 119.3, 117.3, 117.1, 97.4, 80.1, 80.0, 77.6, 77.2, 69.4, 68.6, 67.9, 67.2, 45.7, 41.3, 39.9, 36.6, 35.8, 35.5, 34.2, 31.2, 28.0, 27.4, 25.8, 19.9, 19.2, 17.4, 14.6, 12.5, 11.7, -4.6.

To a solution of (**i**) (10.0 g, 14 mmoL) and N, N-diisopropylethylamine (DIPEA, 4.43g, 68 mmoL) in DCM (300 mL) at 0 °C was added chloromethyl methyl ether (MOMCl, 2.53 g, 62 mmoL), and the reaction was allowed to reach ambient temperature and stirred for 3 h. TLC was utilised to monitor the reaction. After washing with water (100 mL) and brine (100 mL), the organic layer was dried over anhydrous MgSO_4_, filtered, concentrated *in vacuo*, and purified with silica gel chromatography (EtOAc/hexane = 1:5) to yield (**ii**) (9.7 g, 91%). ^1^H NMR (600 MHz, CDCl_3_): δ_H_ 5.78 (m, 1H), 5.71 (m, 1H), 5.70 (m, 1H), 5.32 (m, 1H), 5.26 (m, 1H), 5.18 (m, 1H), 4.67 (dd, *J* = 14.0, *J* = 2.0 Hz, 1H), 4.59 (m, 2H), 4.57 (dd, *J* = 14.0, *J* = 2.0 Hz, 1H), 4.43 (br, 1H), 4.11 (s, 1H), 3.89 (br, 1H), 3.81 (d, *J* = 6.0 Hz, 1H), 3.67 (m, 1H), 3.42 (s, 3H), 3.34 (m, 1H), 3.18 (d, *J* = 8.8 Hz, 1H), 2.53 (m, 1H), 2.31 (m, 2H), 1.98 (m, 1H), 1.78 (s, 3H), 1.74 (m, 1H), 1.64 (m, 1H), 1.53 (m, 1H), 1.51 (s, 3H), 1.51 (m, 2H), 1.49 (m, 1H), 1.47 (m, 1H), 1.42 (m, 2H), 1.31 (m, 1H), 1.16 (d, *J* = 7.2 Hz, 3H), 0.95 (t, *J* = 7.2 Hz, 3H), 0.92 (s, 9H), 0.83 (d, *J* = 6.6 Hz, 3H), 0.82 (m, 1H), 0.78 (d, *J* = 6.6 Hz, 3H), 0.12 (s, 6H); ^13^C NMR (150 MHz, CDCl_3_): δ_C_ 173.8, 140.2, 137.5, 137.3, 135.2, 124.7, 119.3, 118.3, 117.3, 97.4, 95.5, 82.6, 80.1, 80.1, 77.2, 69.5, 68.6, 67.9, 67.2, 56.1, 45.7, 41.3, 39.7, 36.8, 35.8, 35.5, 34.3, 31.2, 28.0, 27.4, 25.9, 20.0, 19.6, 17.4, 15.0, 12.5, 11.7, -4.6.

TsOH (2 g) was dissolved in methanol (200 mL), followed by the addition of (**ii**) (10.8 g, 14.5 mmoL), and the reaction was stirred at ambient temperature overnight. TLC was utilised to monitor the reaction. After removal of solvent *in vacuo*, the residue was purified by flash column chromatography (EtOAc/hexane = 1:2) to yield **IVM-2** (7.9 g, 86%). ^1^H NMR (600 MHz, CDCl_3_), see Figure S6: δ_H_ 5.70 (m, 1H), 5.70 (m, 1H), 5.40 (br, 1H), 5.30 (m, 1H), 5.18 (d, *J* = 12.0 Hz, 1H), 4.68 (dd, *J* = 14.0, *J* = 2.0 Hz, 1H), 4.64 (dd, *J* = 14.0, *J* = 2.0 Hz, 1H), 4.59 (m, 2H), 4.28 (br, 1H), 4.08 (s, 1H), 3.95 (d, *J* = 6.0 Hz, 1H), 3.89 (br, 1H), 3.67 (m, 1H), 3.42 (s, 3H), 3.25 (m, 1H), 3.18 (d, *J* = 8.8 Hz, 1H), 2.52 (m, 1H), 2.38 (d, *J* = 7.2 Hz, 1H), 2.28 (m, 2H), 1.97 (m, 1H), 1.86 (s, 3H), 1.75 (m, 1H), 1.65 (m, 1H), 1.53 (m, 1H), 1.50 (s, 3H), 1.50 (m, 2H), 1.50 (m, 1H), 1.47 (m, 1H), 1.42 (m, 2H), 1.31 (m, 1H), 1.16 (d, *J* = 7.2 Hz, 3H), 0.95 (t, *J* = 7.2 Hz, 3H), 0.84 (d, *J* = 6.6 Hz, 3H), 0.80 (m, 1H), 0.78 (d, *J* = 6.6 Hz, 3H); ^13^C NMR (150 MHz, CDCl_3_), see Figure S7: δ_C_ 173.6, 139.7, 138.0, 137.7, 135.1, 124.6, 120.4, 118.3, 118.1, 97.6, 95.5, 82.6, 80.2, 79.1, 77.2, 68.5, 68.5, 67.7, 67.2, 56.1, 45.7, 41.3, 39.8, 36.8, 35.8, 35.5, 34.2, 31.2, 28.0, 27.4, 19.9, 19.5, 17.4, 14.9, 12.5, 11.7. HRESIMS *m*/*z* 653.3661 [M + Na] ^+^ (calcd for C_36_H_54_O_9_Na, 653.3660), see Figure S8.

**Preparation of IVM-3**

To a solution of **IVM-2** (500 mg, 0.8 mmoL), triphenylphosphine (1250 mg, 4.8 mmoL) and formic acid (220 mg, 4.8 mmoL) in THF (30 mL) at ambient temperature was added diisopropyl azodicarboxylate (DIAD, 962 mg, 4.8 mmoL), and the reaction was stirred overnight. TLC was utilised to monitor the reaction. After removal of the solvent *in vacuo*, the residue was purified by flash column chromatography (EtOAc/hexane = 1:4) to yield (**iii**) (422 mg, 81%). ^1^H NMR (600 MHz, CDCl_3_): δ_H_ 8.18 (s, 1H), 5.89 (m, 1H), 5.72 (m, 1H), 5.71 (m, 1H), 5.62 (br, 1H), 5.40 (br, 1H), 5.35 (m, 1H), 5.18 (m, 1H), 4.60 (d, *J* = 7.2 Hz, 1H), 4.60 (br, 2H), 4.57 (d, *J* = 7.2 Hz, 1H), 3.89 (br, 1H), 3.75 (s, 1H), 3.68 (m, 1H), 3.43 (s, 3H), 3.20 (d, *J* = 9.0 Hz, 1H), 3.08 (m, 1H), 2.53 (m, 1H), 2.32 (m, 1H), 2.27 (m, 1H), 1.97 (m, 1H), 1.78 (s, 3H), 1.77 (m, 1H), 1.64 (m, 1H), 1.54 (m, 1H), 1.51 (m, 1H), 1.50 (s, 3H), 1.50 (m, 2H), 1.47 (m, 1H), 1.43 (m, 2H), 1.34 (m, 1H), 1.16 (d, *J* = 7.2 Hz, 3H), 0.96 (t, *J* = 7.2 Hz, 3H), 0.85 (d, *J* = 6.6 Hz, 3H), 0.80 (m, 1H), 0.79 (d, *J* = 6.6 Hz, 3H); ^13^C NMR (150 MHz, CDCl_3_): δ_C_ 173.1, 160.3, 138.2, 138.1, 135.2, 133.2, 124.8, 121.7, 120.1, 118.3, 97.4, 95.5, 83.9, 82.6, 77.3, 75.8, 70.3, 68.6, 68.1, 67.3, 56.2, 46.3, 41.3, 40.0, 36.8, 35.8, 35.5, 34.2, 31.2, 28.0, 27.4, 19.5, 18.9, 17.5, 14.9, 12.5, 11.7.

To a solution of (**iii**) (345 mg, 0.52 mmoL) in THF/MeOH/H_2_O (10/5/0.5 mL) at ambient temperature was added sodium bicarbonate (88 mg, 1.05 mmoL), and the reaction was stirred overnight. TLC was utilised to monitor the reaction. The mixture was extracted with DCM (3 × 15 mL). The combined organic layers were washed with brine, dried over anhydrous MgSO_4_, filtered, concentrated *in vacuo*, and purified with silica gel chromatography (EtOAc/hexane = 1:2) to yield **IVM-3** (313 mg, 95%). ^1^H NMR (600 MHz, CDCl_3_), see Figure S9: δ_H_ 5.85 (m, 1H), 5.70 (m, 1H), 5.70 (m, 1H), 5.42 (br, 1H), 5.34 (m, 1H), 5.18 (m, 1H), 4.59 (m, 2H), 4.59 (m, 2H), 4.12 (br, 1H), 4.01 (br, 1H), 3.89 (br, 1H), 3.83 (br, 1H), 3.67 (m, 1H), 3.42 (s, 3H), 3.19 (d, *J* = 9.0 Hz, 1H), 3.04 (m, 1H), 2.52 (m, 1H), 2.32 (m, 1H), 2.26 (m, 1H), 1.97 (m, 1H), 1.90 (s, 3H), 1.76 (m, 1H), 1.64 (m, 1H), 1.53 (m, 1H), 1.50 (m, 1H), 1.50 (m, 2H), 1.50 (s, 3H), 1.47 (m, 1H), 1.42 (m, 2H), 1.32 (m, 1H), 1.16 (d, *J* = 7.2 Hz, 3H), 0.95 (t, *J* = 7.2 Hz, 3H), 0.85 (d, *J* = 6.6 Hz, 3H), 0.80 (m, 1H), 0.78 (d, *J* = 6.6 Hz, 3H); ^13^C NMR (150 MHz, CDCl_3_), see Figure S10: δ_C_ 173.7, 139.2, 137.8, 137.4, 135.1, 124.8, 120.9, 118.3, 117.0, 97.4, 95.5, 85.7, 82.6, 77.3, 76.0, 69.4, 68.5, 68.2, 67.3, 56.2, 46.5, 41.3, 39.9, 36.9, 35.8, 35.5, 34.2, 31.2, 28.0, 27.4, 19.6, 19.3, 17.5, 14.9, 12.5, 11.7. HRESIMS *m*/*z* 653.3660 [M + Na] ^+^ (calcd for C_36_H_54_O_9_Na, 653.3660), see Figure S11.

**Preparation of bdpy**

To a suspension of 4-hydroxybenzaldehyde (3.66 g, 30 mmoL) and potassium carbonate (16 g, 120 mmoL) in acetone (250 mL) was added propargyl bromide (14.3 g, 120 mmoL), and the reaction was stirred at ambient temperature overnight. TLC was utilised to monitor the reaction. After filtration, the filtrate was evaporated *in vacuo* and the residue was partitioned between EtOAc (200 mL) and water (200 mL). The organic layer was washed with brine, dried over MgSO_4_, filtered, concentrated *in vacuo* to yield, and purified with silica gel chromatography (EtOAc/hexane = 1:4) to yield (**iv**) (4.2 g, 88%). ^1^H NMR (600 MHz, CDCl_3_): δ_H_ = 9.91 (s, 1H), 7.86 (d, *J* = 8.0 Hz, 2H), 7.09 (d, *J* = 8.0 Hz, 2H), 4.78 (s, 2H), 2.57 (s, 1H); ^13^C NMR (150 MHz, CDCl_3_): δ_C_ = 190.8, 162.4, 131.9, 130.6, 115.2, 77.5, 76.4, 55.9.

To a solution of (**iv**) (230 mg, 1.44 mmoL) and 3-ethyl-2, 4-dimethylpyrrole (**v**) (407 mg, 3.3 mmoL) in DCM (15 mL) was added TFA (110 mg, 0.96 mmoL). The dark mixture was stirred at r.t. until total disappearance of (**iv**) (TLC). Then DDQ (523 mg, 2.3 mmoL) was added, and 5 min later TEA (1170 mg, 11.5 mmoL) and trifluoroborate etherate (2450 mg, 17.25 mmoL) were added successively. TLC was utilised to monitor the reaction. The mixture was filtered through a pad of silica. After removal of solvent *in vacuo*, the residue was purified by flash column chromatography (EtOAc/hexane = 1:15) to yield **bdpy** (130 mg, 20.8% over 3 steps). ^1^H NMR (600 MHz, CDCl_3_): δ_H_ = 7.19 (d, *J* = 8.0 Hz, 2H), 7.08 (d, *J* = 8.0 Hz, 2H), 4.76 (s, 2H), 2.56 (t, *J* = 2.0 Hz, 1H), 2.53 (s, 6H), 2.30 (q, *J* = 7.6 2H), 1.33 (s, 6H), 0.98 (t, *J* = 7.6 Hz, 3H); ^13^C NMR (150 MHz, CDCl_3_): δ_C_ = 158.0, 153.6, 139.9, 138.4, 132.7, 131.1, 129.5, 128.9, 115.5, 78.1, 75.9, 56.0, 17.1, 14.6, 12.5, 11.8.

**Preparation of IVM-bdpy**

A solution of acetone/H_2_O (30 mL/10 mL) of ethyl 4-bromobutyrate (**vi**) (3.9 g, 20 mmoL) and sodium azide (2.6 g, 40 mmoL) was heated under reflux overnight. Then acetone was removed *in vacuo* and the residue was partitioned between DCM (30 mL) and water (30 mL). The mixture was extracted with DCM (3 × 20 mL), and the combined organic extracts were washed with water, brine, dried over MgSO_4_ and evaporated to dryness to give the azido ester as colourless oil. The obtained azido acid ethyl ester was dissolved in MeOH/water (30 mL/30 mL) solution, cooled to 0 °C and KOH (2.2 g, 40 mmol) was added. The reaction mixture was stirred at room temperature overnight. The methanol was removed *in vacuo* and the residue was partitioned between DCM (30 mL) and water (30 mL). The aqueous layer was extracted with DCM (2 × 20 mL), acidified to pH 1 with 1N aqueous HCl and extracted with EtOAc (4 × 30 mL). The combined organic layers were dried over MgSO_4_ and evaporated to dryness to afford (**vii**) (2.2 g, 85%) as colorless oil. To a solution of (**vii**) (500 mg, 3.88 mmoL) in DCM (10 mL) was added oxalyl chloride (1500 mg, 11.6 mmoL) at ambient temperature, followed by the addition of 3 drops of DMF. The mixture was stirred for another 3 h, and then the solvent was removed under N_2_ to yield (**viii**), which was used without further purification.

To a solution of (**i**) (1360 mg, 1.94 mmoL), TEA (589 mg, 5.82 mmoL) and DMAP (710 mg, 5.82 mmoL) in DCM (20 mL) at ambient temperature was added (**viii**) (reaction mixture, 3.88 mmoL), and the reaction was stirred overnight. TLC was utilised to monitor the reaction. The mixture was poured into water (25 mL) and extracted with DCM (3 × 20 mL). The combined organic layers were washed with brine, dried over anhydrous MgSO_4_, filtered, concentrated *in vacuo* to yield, and purified with silica gel chromatography (EtOAc/hexane = 1:4) to yield (**ix**) (1200 mg, 76%). ^1^H NMR (600 MHz, CDCl_3_): δ_H_ 5.81 (m, 1H), 5.77 (m, 1H), 5.67 (m, 1H), 5.32 (br, 1H), 5.30 (m, 1H), 5.16 (br, 1H), 4.98 (m, 1H), 4.68 (d, *J* = 7.2 Hz, 1H), 4.58 (d, *J* = 7.2 Hz, 1H), 4.44 (m, 1H), 4.17 (s, 1H), 3.82 (d, *J* = 5.6 Hz, 1H), 3.63 (m, 1H), 3.39 (t, *J* = 6.6 Hz, 2H), 3.37 (br, 1H), 3.19 (d, *J* = 9.0 Hz, 1H), 2.64 (m, 1H), 2.53 (m, 2H), 2.26 (m, 2H), 1.99 (m, 1H), 1.96 (m, 2H), 1.79 (s, 3H), 1.71 (m, 1H), 1.64 (m, 1H), 1.58 (s, 3H), 1.55 (m, 1H), 1.51 (m, 1H), 1.51 (m, 2H), 1.47 (m, 1H), 1.43 (m, 2H), 1.33 (m, 1H), 1.03 (d, *J* = 7.2 Hz, 3H), 0.95 (t, *J* = 7.2 Hz, 3H), 0.93 (s, 9H), 0.85 (d, *J* = 6.6 Hz, 3H), 0.84 (m, 1H), 0.78 (d, *J* = 6.6 Hz, 3H), 0.13 (s, 6H); ^13^C NMR (150 MHz, CDCl_3_): δ_C_ 173.9, 171.8, 141.2, 137.5, 136.2, 134.6, 125.4, 119.0, 117.7, 117.3, 97.5, 80.2, 80.1, 78.9, 76.9, 69.4, 68.7, 67.9, 67.1, 50.6, 45.7, 41.3, 38.9, 36.7, 35.8, 35.5, 34.2, 31.2, 31.1, 28.0, 27.3, 25.9, 24.3, 20.0, 18.9, 18.4, 17.4, 14.7, 12.5, 12.0, -4.6.

TsOH (100 mg) was dissolved in methanol (10 mL), followed by the addition of **ix** (522 mg, 1.94 mmoL), and the reaction was stirred at ambient temperature overnight. TLC was utilised to monitor the reaction. After removal of solvent *in vacuo*, the residue was purified by flash column chromatography (EtOAc/hexane = 1:2) to yield (**x**) (350 mg, 78%). ^1^H NMR (600 MHz, CDCl_3_): δ_H_ 5.86 (m, 1H), 5.78 (m, 1H), 5.68 (m, 1H), 5.42 (br, 1H), 5.33 (m, 1H), 5.16 (br, 1H), 4.98 (m, 1H), 4.70 (d, *J* = 7.2 Hz, 1H), 4.65 (d, *J* = 7.2 Hz, 1H), 4.29 (d, *J* = 6.0 Hz, 1H), 3.97 (d, *J* = 6.6 Hz, 1H), 3.63 (m, 1H), 3.39 (t, *J* = 6.6 Hz, 2H), 3.27 (m, 1H), 3.19 (d, *J* = 9.0 Hz, 1H), 2.64 (m, 1H), 2.53 (m, 2H), 2.25 (m, 2H), 1.98 (m, 1H), 1.95 (m, 2H), 1.87 (s, 3H), 1.72 (m, 1H), 1.64 (m, 1H), 1.57 (s, 3H), 1.55 (m, 1H), 1.51 (m, 1H), 1.50 (m, 2H), 1.47 (m, 1H), 1.43 (m, 2H), 1.34 (m, 1H), 1.04 (d, *J* = 7.2 Hz, 3H), 0.95 (t, *J* = 7.2 Hz, 3H), 0.85 (d, *J* = 6.6 Hz, 3H), 0.82 (m, 1H), 0.78 (d, *J* = 6.6 Hz, 3H); ^13^C NMR (150 MHz, CDCl_3_): δ_C_ 173.6, 171.8, 140.6, 137.9, 136.7, 134.5, 125.3, 120.1, 118.0, 117.7, 97.5, 80.3, 79.1, 78.8, 76.9, 68.6, 68.4, 67.7, 67.0, 50.6, 45.6, 41.2, 39.0, 36.8, 35.7, 35.5, 34.1, 31.2, 31.1, 28.0, 27.3, 24.3, 19.9, 18.8, 17.4, 14.6, 12.5, 12.0.

A mixture of (**x**) (69.7 mg, 0.1 mmoL), bpdy (43 mg, 0.1 mmoL), copper sulfate pentahydrate (2.5 mg, 0.01 mmoL) and sodium ascorbate (2.0 mg, 0.01 mmoL) in acetonitrile (10 mL) was heated to 82 °C for 5 days under nitrogen. TLC were utilised to monitor the reaction. After removal of solvent *in vacuo*, the residue was purified by flash column chromatography (EtOAc/hexane = 1:1) to yield **IVM-bdpy** (81 mg, 71%). ^1^H NMR (600 MHz, CD_3_OD), see Figure S12: δ_H_ 8.10 (s, 1H), 7.19 (s, 1H), 7.19 (s, 1H), 5.95 (m, 1H), 5.81 (m, 1H), 5.67 (m, 1H), 5.42 (s, 1H), 5.24 (s, 2H), 5.20 (br, 1H), 5.13 (m, 1H), 5.00 (m, 1H), 4.65 (d, *J* = 7.2 Hz, 1H), 4.58 (d, *J* = 7.2 Hz, 1H), 4.52 (t, *J* = 7.2 Hz, 2H), 4.23 (m, 1H), 3.77 (d, *J* = 6.0 Hz, 1H), 3.68 (m, 1H), 3.24 (m, 1H), 3.23 (s, 1H), 2.78 (m, 1H), 2.51 (q, *J* = 7.2 Hz, 2H), 2.46 (s, 3H), 2.34 (q, *J* = 7.8 Hz, 2H), 2.25 (m, 2H), 2.25 (m, 2H), 2.20 (m, 1H), 1.89 (m, 1H), 1.82 (s, 3H), 1.60 (m, 1H), 1.59 (s, 3H), 1.56 (m, 1H), 1.51 (m, 1H), 1.48 (m, 2H), 1.46 (m, 1H), 1.41 (m, 2H), 1.35 (s, 3H), 1.19 (m, 1H), 1.05 (d, *J* = 7.2 Hz, 3H), 0.98 (t, *J* = 7.8 Hz, 3H), 0.93 (t, *J* = 7.2 Hz, 3H), 0.86 (d, *J* = 6.6 Hz, 3H), 0.83 (m, 1H), 0.78 (d, *J* = 6.6 Hz, 3H); ^13^C NMR (150 MHz, CD_3_OD), see Figure S13: δ_C_ 173.3, 173.1, 160.4, 154.6, 144.8, 142.5, 142.0, 139.7, 137.1, 137.1, 136.2, 134.0, 132.3, 130.8, 129.5, 127.1, 125.4, 121.4, 120.1, 118.9, 116.8, 98.9, 82.1, 81.8, 80.3, 77.9, 70.1, 68.9, 68.7, 68.6, 62.6, 50.5, 47.0, 42.8, 40.3, 37.7, 36.8, 36.8, 35.0, 32.5, 31.6, 29.2, 28.4, 26.7, 19.8, 19.4, 17.8, 17.8, 15.1, 15.1, 13.0, 12.6, 12.5, 12.2. HRESIMS *m*/*z* 1154.6172 [M + Na] ^+^ (calcd for C_64_H_84_BF_2_N_5_O_10_Na, 1154.6172), see Figure S14.

**Figure S1.** ^1^H NMR (DMSO-*d*_6_, 600 MHz) spectra of IVM

**Figure S2.** ^13^C NMR (DMSO-*d*_6_, 150 MHz) spectra of IVM

**Figure S3.** ^1^H NMR (CDCl_3_, 600 MHz) spectra of IVM-1

**Figure S4.** ^13^C NMR (CDCl_3_, 150 MHz) spectra of IVM-1

**
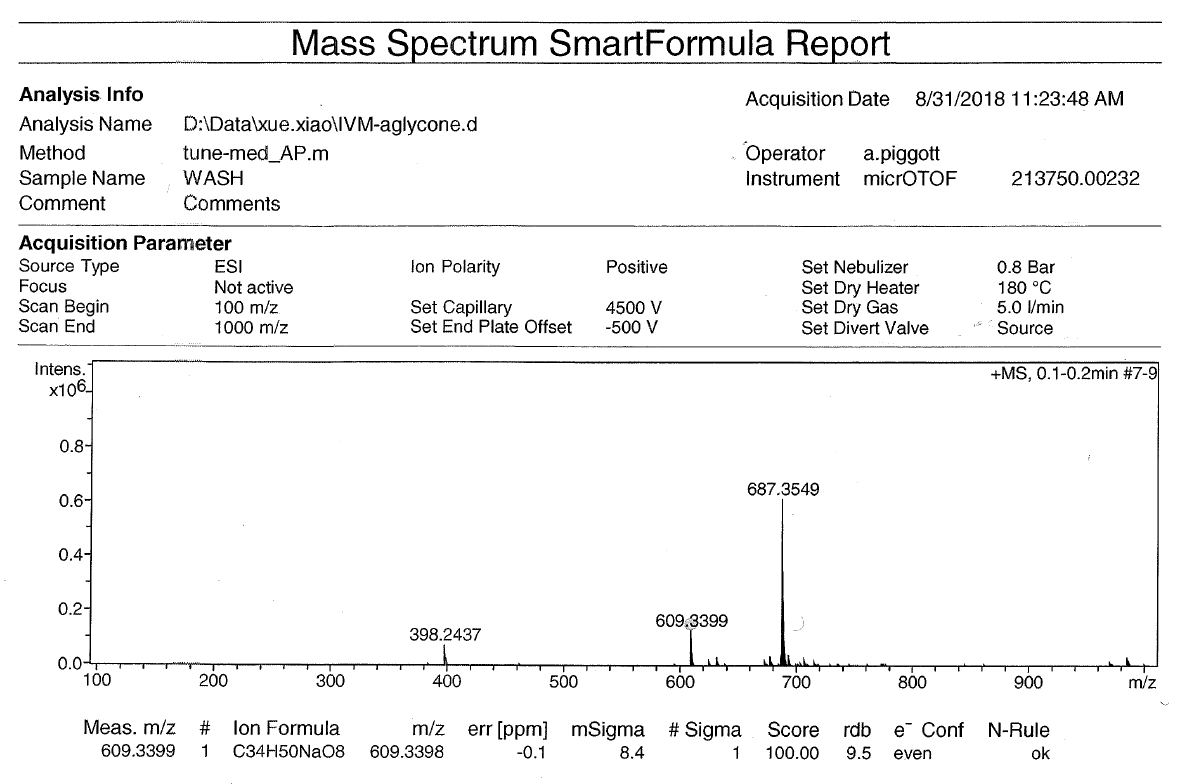
**

**Figure S5.** HRMS data for IVM-1

**Figure S6.** ^1^H NMR (CDCl_3_, 600 MHz) spectra of IVM-2

**Figure S7.** ^13^C NMR (CDCl_3_, 150 MHz) spectra of IVM-2

**
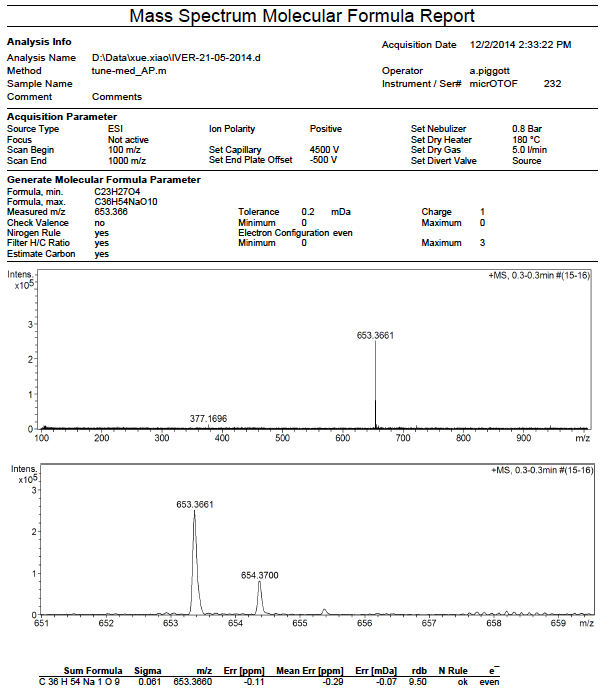
**

**Figure S8**. HRMS data for IVM-2

**Figure S9.** ^1^H NMR (CDCl_3_, 600 MHz) spectra of IVM-3

**Figure S10.** ^13^C NMR (CDCl_3_, 150 MHz) spectra of IVM-3

**
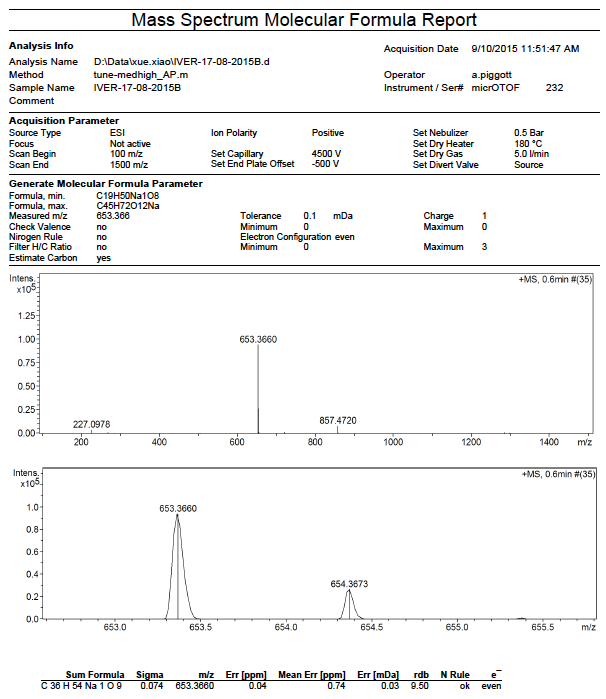
**

**Figure S11.** HRMS data for IVM-3

**Figure S12.** ^1^H NMR (MeOH-*d*_4_, 600 MHz) spectra of IVM-bdpy

**Figure S13.** ^13^C NMR (MeOH-*d*_4_, 150 MHz) spectra of IVM-bdpy


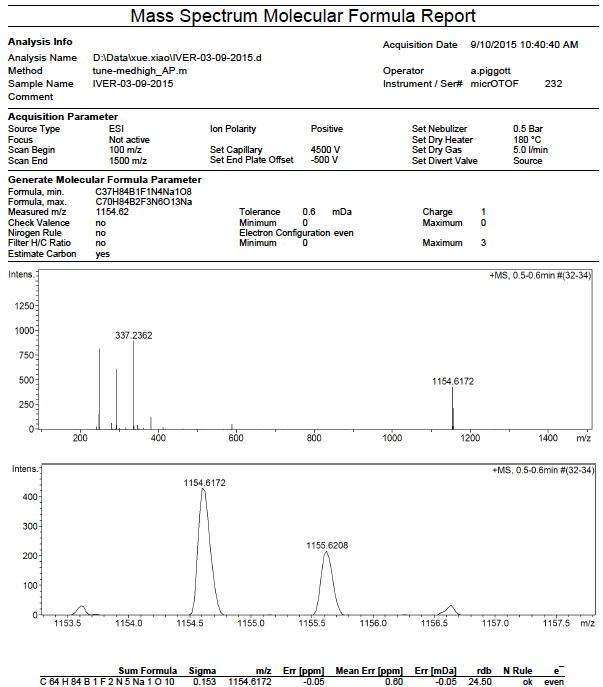


**Figure S14.** HRMS data for IVM-bdpy
